# The Role of *EjSPL3*, *EjSPL4*, *EjSPL5,* and *EjSPL9* in Regulating Flowering in Loquat (*Eriobotrya* *japonica* Lindl.)

**DOI:** 10.3390/ijms21010248

**Published:** 2019-12-30

**Authors:** Yuanyuan Jiang, Jiangrong Peng, Man Wang, Wenbing Su, Xiaoqing Gan, Yi Jing, Xianghui Yang, Shunquan Lin, Yongshun Gao

**Affiliations:** 1 State Key Laboratory for Conservation and Utilization of Subtropical Agro-bioresources, College of Horticulture, South China Agricultural University, Wushan Road 483, Tianhe District, Guangzhou 510642, China; yyjiang613@163.com (Y.J.); jiangrongp@163.com (J.P.); wm014527@163.com (M.W.); suwenbing13@163.com (W.S.); ganxq0702@163.com (X.G.); gzyxh@scau.edu.cn (X.Y.); 2 BGI Genomics, BGI-Shenzhen, Shenzhen 518083, China; shenqiandelan@live.cn; 3 Beijing Academy of Forestry and Pomology Sciences, Beijing Academy of Agriculture and Forestry Sciences, Beijing 100093, China

**Keywords:** loquat, SPL transcription factor, flowering time, aging pathway

## Abstract

The age pathway is important for regulating flower bud initiation in flowering plants. The major regulators in this pathway are miR156 and *SPL* transcription factors. To date, *SPL* genes have been identified in many species of plants. Loquat, as a woody fruit tree of Rosaceae, is unique in flowering time as it blooms in winter. However, the study of its *SPL* homologous genes on the regulation mechanism of flowering time is still limited. In this study, four *SPL* homologs—*EjSPL3*, *EjSPL4*, *EjSPL5,* and *EjSPL9*—are cloned from loquat, and phylogenetic analysis showed that they share a high sequence similarity with the homologues from other plants, including a highly conserved SQUAMOSA promoter binding protein (SBP)-box domain. EjSPL3, EjSPL4, EjSPL5 are localized in the cytoplasm and nucleus, and EjSPL9 is localized only in the nucleus. EjSPL4, EjSPL5, and EjSPL9 can significantly activate the promoters of *EjSOC1-1*, *EjLFY-1,* and *EjAP1-1*; overexpression of *EjSPL3*, *EjSPL4*, *EjSPL5,* and *EjSPL9* in wild-type *Arabidopsis thaliana* can promote flowering obviously, and downstream flowering genes expression were upregulated. Our work indicated that the EjSPL3, EjSPL4, EjSPL5, and EjSPL9 transcription factors are speculated to likely participate in flower bud differentiation and other developmental processes in loquat. These findings are helpful to analyze the flowering regulation mechanism of loquat and provide reference for the study of the flowering mechanism of other woody fruit trees.

## 1. Introduction

Flowering is regulated by extensive factors, such as plant hormones, photoperiod, vernalization, age, temperature, light quality, sucrose, etc. [1,2,3,4,5,6]. Genes regulating flowering time have been characterized in many species, and they interact to form a complicated regulatory network [7,8,9]. *FLOWERING LOCUS T* (*FT*) is a crucial integrator in this network [10], and the flowering time regulating gene *SUPPRESSOR OF OVEREXPRESSION OF CO1* (*SOC1*), the floral meristem identity genes *APETALA1* (*AP1*), *LEAFY* (*LFY*), and *FRUITFULL* (*FUL*) are located at downstream of the regulatory network [11,12]. The aging pathway is reported to be a universal approach to control the transition from vegetative to reproductive stage, and it is primarily coordinated by microRNA156 (miR156) and its target genes *SQUAMOSA PROMOTER BINDING-LIKE* (*SPL*) transcription factors [13,14]. MicroRNAs (miRNAs) are non-coding, small-molecule RNAs of about 16–29 nt in length, are ubiquitous in organisms, and they could regulate gene expression by mediating targeted mRNA degradation or translational repression [15,16]. The aging pathway mainly involves miR156 and miR172 [14]. The targets of miR156 are the *SPL* transcription factors which were first discovered and confirmed in *Antirrhinum majus*, and were named *SQUAMOSA PROMOTER BINDING PROTEIN* (*SBP*) because they recognize and bind to the promoter region of *MADS-box* genes *SQUAMOSA* (*SQUA*) [17]. *SBP* genes were also found in green plants and referred to as the *SQUAMOSA PROMOTER BINDING-LIKE* (*SPL*) genes [18]. In recent decades, the *SBP* gene families were characterized in model plants, woods, crops, and fruits, such as *Arabidopsis thaliana* [19], barley [20], green algae [21], moss [22], tomato [23], salvia [24], rice [25], birch [26], light birch [27], cotton [28], grape [29], apple [30], corn [31], and so on. All *SPL* genes contain a highly conserved DNA binding domain, which was called the SBP-box domain, and it consists of approximately 80 amino acid residues including two zinc finger structures and one nuclear localization signal [32].

The aging pathway regulates flowering under uninduced conditions, and also, it can be integrated with other pathways [9]. In young *Arabidopsis thaliana*, miR156 is in a higher expression level, which negatively regulates the target *SPL*s; as the plant grows, the expression level of miR156 decreases, and the target *SPL*s mRNA increases subsequently [13]. Among the *SPL*s, *SPL9* and *SPL15* positively regulate miR172, which inhibits the expression of transcription factor *APETELA2* (*AP2*); *AP2* inhibits the expression of *FT* and; therefore, delays flowering ultimately [33,34]. At the same time, SPL2, SPL3, SPL4, SPL5, SPL9, SPL10, SPL11, SPL13, and SPL15 can directly promote the expression of *AP1*, *LFY,* and *FUL*, and SPL3, SPL4, and SPL5 can also recruit FT-FD complex and bind to the promoter regions of *AP1*, *LFY* and *FUL* and consequently initiate their transcription [13,35]. Furthermore, SPL2, SPL9, SPL10, SPL11, SPL13, and SPL15 directly regulate *SUPPRESSOR OF OVEREXPRESSION OF CONSTANS 1* (*SOC1*), and then activate the expression of *AP1*, *LFY,* and *FUL*. *SOC1* also negatively regulates *SPL3*, *SPL4,* and *SPL5* to form a feedback loop [13]. At present, the functions of *SPL* genes in more and more species has been unearthed: Grape *VpSBP11* can regulate floral transition and leaf development [36]; *BlSPLs* in *Betula luminifera* interact with DELLA protein to participate gibberellin regulation [27]. It is reported that, except flowering, *SPL* genes are vital in also other physiological processes in plants, such as adventitious root development [13,37], fertility [38], fruit growth and quality [39], floral organ and shoot development [40,41], plant hormone response [42], morphological differentiation [43], and stresses response [44].

Loquat (*Eriobotrya japonica* Lindl.) is a subtropical evergreen fruit that belongs to Maloideae subfamily of Rosaceae. Compared to its relative fruit trees like apple, pear, and peach, loquat has unique habits, in that it blooms in winter and fruits mature in late spring or early summer [45]. To date, some crucial flowering-related genes from loquat have been cloned. *EjTFL1* and *EjLFY* were identified earliest, and *EjAP1* was characterized later which was found to promote flowering [46,47]. Depending on the genome sequencing data of loquat (from our unpublished information), wild loquat flowering related genes of *EdFT*, *EdFD*, *EdCO,* and *EdGI* were separated and were identified to be related to flowering regulation [48,49]. Most recently, we found that *EjSOC1*s played a vital role in promoting flowering in cultivated loquat [50]. However, the information of the upstream transcription factors participating in loquat flowering regulation is still limited.

In this work, cultivated loquat was employed as research material. The *SPL* homologous genes of *EjSPL3*, *EjSPL4*, *EjSPL5,* and *EjSPL9* were isolated from “Jiefangzhong” (JFZ). The spatiotemporal expression of the *EjSPLs* were analyzed, and their subcellular localization were observed in tobacco leaves. In addition, the interactions between EjSPLs and other flowering proteins or the downstream flowering gene promoters were detected. Finally, because of the transform system has not been built in loquat, the genetic functions of the *EjSPL*s were verified in *Arabidopsis thaliana*. This work discovered the roles of *EjSPL3*, *EjSPL4*, *EjSPL5,* and *EjSPL9* on flowering regulation, and provides further understanding of the unknown transcription factors attending reproductive growth in cultivated loquat.

## 2. Results

### 2.1. Identification of SPL3, SPL4, SPL5, and SPL9 Homologs from Loquat (Eriobotrya japonica Lindl.)

**Figure 1:**
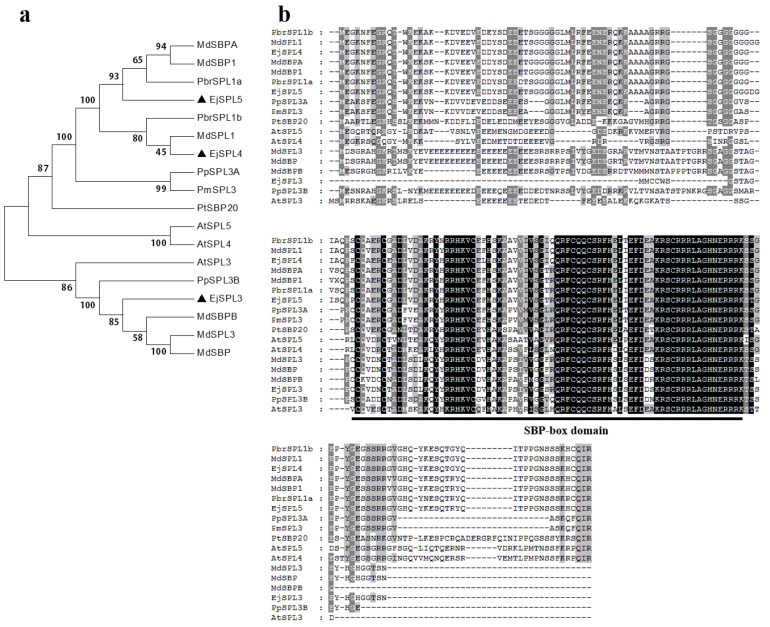
Sequence analysis of SPL3, SPL4, and SPL5 from various species. (**a**) Phylogenetic trees of SPL3, SPL4, and SPL5 in different species. Black triangles indicate loquat EjSPL3, EjSPL4, and EjSPL5. (**b**) Amino acid sequence alignment of SPL3, SPL4, and SPL5. Amino acids with black and gray represent 100% and 50% similarity, respectively; the underline indicates the SBP-box domain. Identical and similar amino acid residues are represented by black and grey shadows, respectively. The accession number of each gene is as follows: *MdSBPA* (*Malus domestica*, ADL36826.1), *MdSBP1* (*Malus domestica*, XP_008386198.1), *PbrSPL1a* (*Pyrus × bretschneideri*, XP_00 9340759.1), *PbrSPL1b* (*Pyrus × bretschneideri*, XP_009356783.1), *MdSPL1* (*Malus domestic*, XP_0083670 70.1), *PpSPL3A* (*Prunus persica*, XP_007213489.1), *PmSPL3* (*Prunus mume*, XP_008226482.1), *PtSBP20* (*Populus tomentosa*, AOF41322.1), *AtSPL5* (AT3G15270), *AtSPL4* (AT1G53160), *AtSPL3* (AT2G33810), *PpSPL3B* (*Prunus persica*, XP_007212177.1), *MdSBPB* (*Malus domestica*, ADL36825.1), *MdSPL3* (*Malus domestica*, XP_0083837 04.1), *MdSBP* (*Malus domestica*, AHC08502.1).

The genes of *EjSPL3*, *EjSPL4*, and *EjSPL5* from the cultivated loquat “JFZ” were annotated within the genome-wide scope of “JFZ” genome (from our unpublished data), and their coding sequences were isolated. *EjSPL3*, *EjSPL4,* and *EjSPL5* had 303, 570, and 576 nucleotides encoding 100, 189, and 191 amino acids, respectively (Sequence S1). Clustering analysis between the predicted amino acid sequences of the above EjSPLs and their homologous genes in other plants was conducted, the phylogenetic tree showed that EjSPL3, EjSPL4, and EjSPL5 were clustered in different branches, and the closest homologs to them were from apple or pear: EjSPL3 and MdSBPB, EjSPL4 and MdSPL1 are the closest, and EjSPL5 is closest to PbrSPL1a (Figure 1a). From the sequence alignment, there was a conserved SBP-box domain in each of them, including two zinc finger structures and a nuclear localization signal (Figure 1b). By blast alignment, EjSPL3 showed 40% and 53% identity compared with AtSPL3 and MdSBPB; EjSPL4 had 51% and 94% identity compared to AtSPL4 and MdSPL1; EjSPL5 had 48% and 96% identity with AtSPL5 and PbrSPL1a, respectively. In conclusion, it is shown that the EjSPLs had higher similarity with those from apple and pear compared to other reported SPLs.

**Figure 2:**
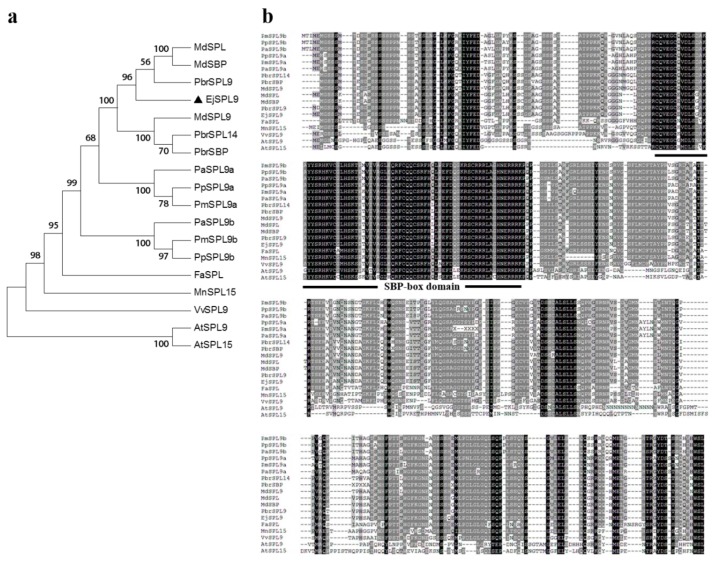
Sequence analysis of SPL9 from various species. (**a**) Phylogenetic tree of SPL9s in different species. Black triangle indicated EjSPL9. (**b**) Amino acid sequence alignment of SPL9s. Amino acids with black and gray represent 100% and 50% similarity, respectively; the underline indicated the SBP-box domain. Identical and similar amino acid residues are represented by black and grey shadows, respectively. The accession number of each gene is as follows: *PbrSPL9* (*Pyrus × bretschneideri*, XP_009369782.1), *MdSPL* (*Malus domestica*, ADL36823.1), *MdSBP* (*Malus domestica*, AHC08503.1), *PbrSPL14* (*Pyrus × bretschneideri*, XP_009376108.1), *MdSPL9* (*Malus domestica*, XP_008392088.1), *PbrSBP* (*Pyrus × bretschneideri*, AIS93133.1), *PaSPL9a* (*Prunus avium*, XP_021830661.1), *PaSPL9b* (*Prunus avium*, XP_021825505.1), *PpSPL9a* (*Prunus persica*, XP_007203426.1), *PmSPL9a* (*Prunus mume*, XP_008240953.1), *PmSPL9b* (*Prunus mume*, XP_008240879.1), *PpSPL9b* (*Prunus persica*, XP_0072053 41.1), *FaSPL* (*Fragaria* × *ananassa*, AEW23126.1), *VvSPL9* (*Vitis vinifera*, NP_001267898.1), *MnSPL15* (*Morus notabilis*, XP_010102560.1), *AtSPL9* (AT2G42200), *AtSPL15* (AT3G57920).

Except *EjSPL3*, *EjSPL4* and *EjSPL5* genes, an *EjSPL9* gene was also isolated from cultivated loquat “JFZ”, and it contained 1140 nucleotides encoding 379 amino acids. The phylogenetic tree showed that EjSPL9 was closely related to SPL9 of pear and farthest from AtSPL9 and AtSPL15 (Figure 2a). From the sequence alignment, the homology of SPL9 is much higher than that of SPL3, SPL4, and SPL5 in woody plants, such as apple, pear, birch, etc., and the similarity is relatively low in herbaceous plants. In addition, they had a highly conserved SBP-box domain also (Figure 2b). By blast alignment, EjSPL9 showed 44% and 96% similarity compared with AtSPL9 and PbrSPL9, respectively.

### 2.2. Analysis of EjSPLs Expression Patterns in Different Tissues

**Figure 3:**
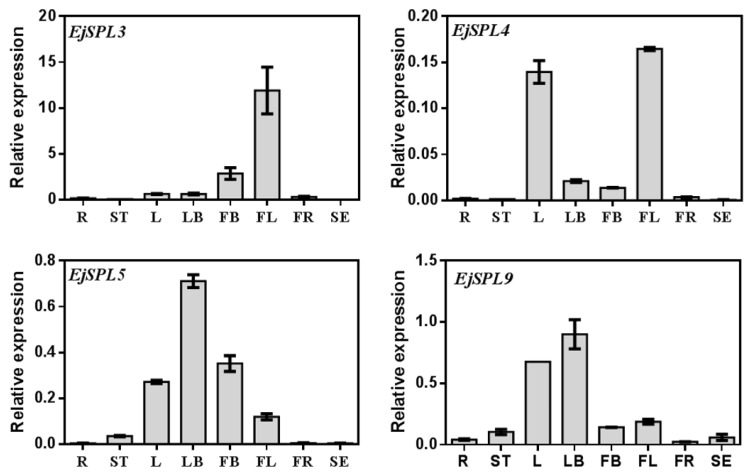
Relative expression levels of *SPL* genes in the different tissues of “JFZ”. The Y-axis represents different tissues, and the X-axis represents the relative expression of the *EjSPL* gene of “JFZ”. R—root, ST—stem, L—mature leaf (2017.4.28), LB—leaf bud (2017.04.28), FB—flower bud (2017.8.18), FL—flower (2017.12.08), FR—fruit (2017.3.17), SE—seed. *Ejβ-actin* as an internal control. Error bars indicating SE from three biological replicates.

In order to identify the role of the *EjSPL3*, *EjSPL4*, *EjSPL5,* and *EjSPL9* in loquat flowering, we analyzed their transcription levels in the samples including leaves, buds, and flowers in different growing periods. Different tissues of the “JFZ” were sampled, including root, stem, mature leaf, leaf bud, flower bud, flower, fruit, and seed. The results showed that all *EjSPL*s were highly expressed in leaves, buds (leaf buds and flower buds), and flowers compared to other tissues. The expression level of *EjSPL4*, *EjSPL5,* and *EjSPL9* in leaf buds was higher than that in flower buds, and *EjSPL3* was expressed relatively higher in flower buds. However, *EjSPL5* and *EjSPL9* were also expressed in stem, and the transcription level of *EjSPL9* was detectable in root and seed (Figure 3). The results suggested that *EjSPL3*, *EjSPL4*, *EjSPL5,* and *EjSPL9* may be involved in the development of leaves, buds, flowers, stems, roots, and seeds.

### 2.3. Expression Pattern of EjSPLs during Growth and Development of Loquat

**Figure 4:**
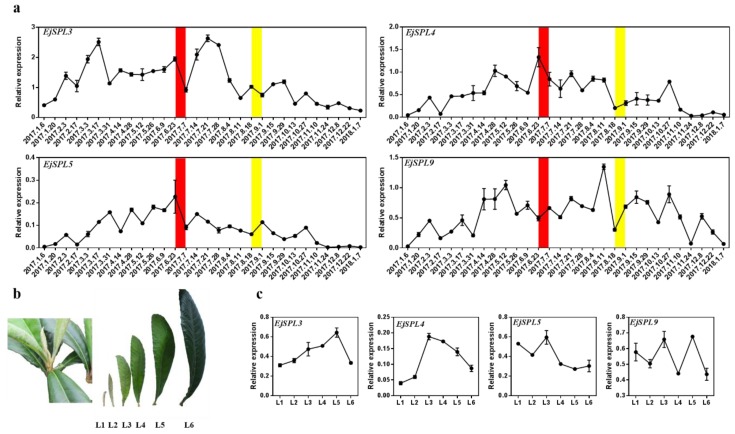
Relative expression levels of *SPL* genes in the leaves of “JFZ”. (**a**) Expression patterns of *SPL* genes in the mature leaves at different periods. (**b**) The leaves at different developmental stages. (**c**) Expression patterns of *SPL* genes in (**b**). Red box and yellow box indicate the beginning of flower bud differentiation and the obvious inflorescence that can be seen, respectively. *Ejβ-actin* as an internal control. Error bars indicating SE from three biological replicates.

To further clarify the roles of *EjSPLs* in loquat, the leaves, buds, and flowers of different developmental stages were sampled for detecting the *EjSPL*s expression levels. In our previous observation of the flower bud paraffin of “JFZ” in Guangzhou [50] the loquat flower bud differentiation happens during the period from 23 June to 7 July, and inflorescence is forming during the end of August to the beginning of September. In the leaves of different periods, the expression of *EjSPL3*, *EjSPL4,* and *EjSPL5* were all in a quite low level at the beginning of the year, and followed by a significant increase till the start of flower bud differentiation (the end of June). Furthermore, the expression level of *EjSPL4* and *EjSPL5* gradually decreased to a low level by the end of the year, similar as that in the beginning of the year. The expression level of *EjSPL9* came to a peak one month earlier than that of *EjSPL4* and *EjSPL5*, and was kept in a relatively high level during the whole period of flower development; it just declined to a very low level after loquat finished flowering. The level of the *EjSPL3* transcripts showed the first peak at the beginning of March, which was even earlier than that of *EjSPL9*, and also it showed another distinct peak during the early stage of flower bud development, and after that, decreased obviously to a low level by the end of the year (Figure 4a).

The expression levels of the *EjSPLs* in the leaves of different developmental stages (Figure 4b) showed that the expression levels of *EjSPL3* and *EjSPL4* were low in the young leaves and increased to a higher level as the leaves developed; after that, they began to decline to a low level again when leaves matured. *EjSPL5* mRNA level was high in the young leaves and started decreasing from L3 stage; *EjSPL9* mRNA level was kept in a relatively high level in all the stages compared to other *EjSPL*s (Figure 4c).

**Figure 5:**
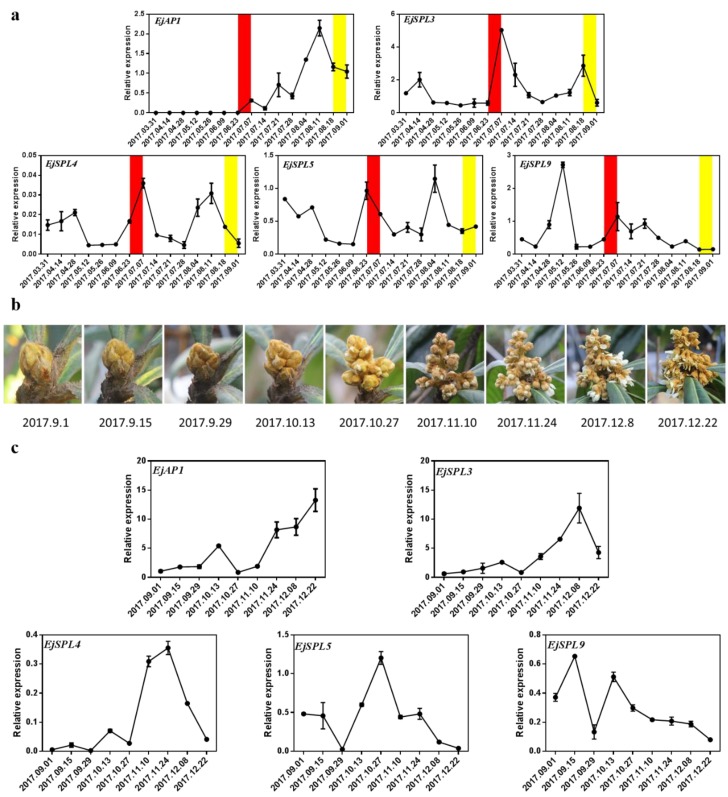
Relative expression levels of *EjSPL* genes in the buds and flowers of “JFZ” at different periods. (**a**) Relative expression levels of *EjSPL* genes in the buds at different periods. (**b**) The flowers in different developmental stages. (**c**) Relative expression levels of *EjSPL* genes in (**b**). Red box and yellow box indicated the beginning of flower bud differentiation and the obvious inflorescence that can be seen, respectively. *Ejβ-actin* as an internal control. Error bars indicating SE from three biological replicates.

*AP1* is a flower meristem identity gene, located in downstream of the flowering regulation network [11], and has a high expression only in shoot apical meristem [50]; so the expression level of *AP1* is generally employed as a marker gene of flowering, especially for confirming the period of floral bud initiation and development. In this study, the expression level of *EjAP1* showed a distinct rise from the end of June, reached the highest level in mid-August, and then declined, which is highly consistent with the period of flower bud differentiation and development in loquat (Figure 5a). In the shoot apical meristem of different periods, the expression levels of *EjSPL3*, *EjSPL4*, and *EjSPL5* all came to a relatively small peak during spring, and compared with them, *EjSPL9* was abundantly expressed at this period. During floral bud initiation, expression levels of all the *EjSPL*s started rising rapidly from 9 or 23 June, got to an obvious peak, which is ten days earlier than that of *EjAP1*, and then decreased immediately; therefore, it is speculated that they may be involved in the process of loquat flower bud differentiation and are upstream of *EjAP1*.

In addition, flower samples were collected from macroscopic flower buds to the start of fruit setting (Figure 5b). As one ABC developmental model gene, the expression level of *EjAP1* was continuously increased in almost all the stages except the end of October. Importantly, *EjSPL3, EjSPL4*, and *EjSPL5* mRNA levels showed similar trend as that of *EjAP1*, and they started decreasing from the end of November or the beginning of December. However, *EjSPL9* showed some different or even opposite expression trends (Figure 5c). Based on these results, we speculated that *EjSPL3*, *EjSPL4*, and *EjSPL5* may be involved in the growing process from the initial floral bud differentiation to the end of flowering, and *EjSPL9* is hypothesized to be important for floral bud differentiation and the early stages of flower organ development.

In summary, these findings suggested that *EjSPL3*, *EjSPL4*, *EjSPL5,* and *EjSPL9* genes were possibly involved in the process of leaf development and flowering (from floral bud initiation to flower development) in loquat.

### 2.4. Subcellular Localization of EjSPL3, EjSPL4, EjSPL5, and EjSPL9

**Figure 6:**
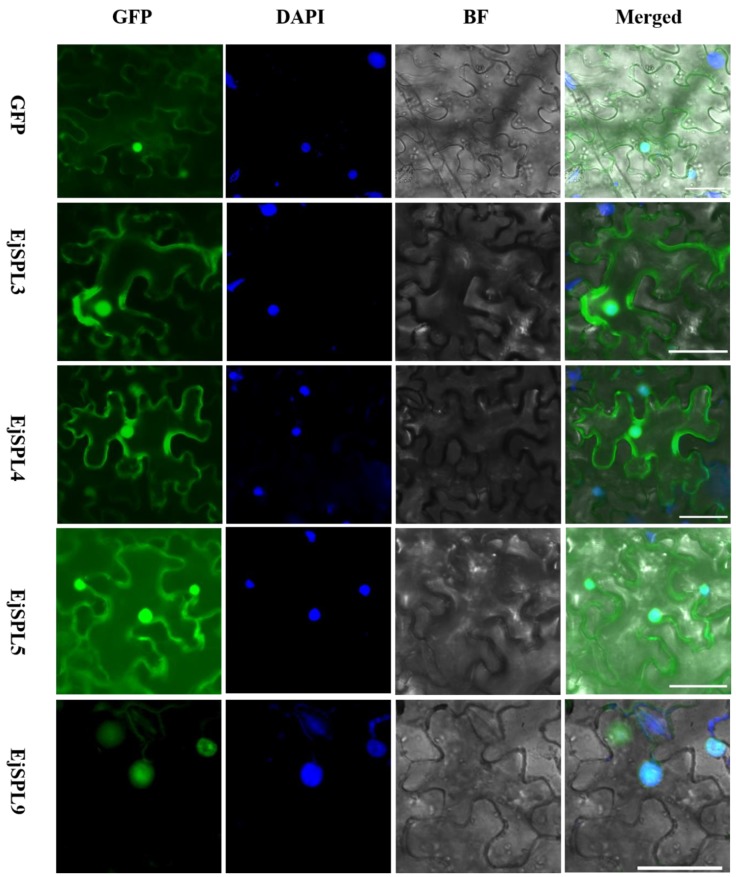
Subcellular localization of EjSPLs in tobacco leaves. GFP, GFP fluorescence channel; 4,6-diamidino-2-phenylindole (DAPI), DAPI fluorescence channel; BF, bright-field; Merged, merged image of GFP, DAPI, and BF. Bars = 50 µm.

In order to verify the localization of *EjSPLs*-encoded proteins in cells, we constructed an EjSPLs fusion expression vector carrying 35S promoter and a green fluorescent protein (GFP)-tagged protein, and introduced them into the epidermal cells of tobacco by *Agrobacterium tumefaciens* infection, and then their fluorescence signals were observed. The results showed that EjSPL3, EjSPL4, and EjSPL5 were localized in the cytoplasm and nucleus, and EjSPL9 was localized only in the nucleus (Figure 6), which suggested that EjSPL3, EjSPL4, EjSPL5, and EjSPL9 may have different functions in loquat.

### 2.5. The regulation of EjSPL3, EjSPL4, EjSPL5, and EjSPL9 on the expression of EjLFY, EjAP1, and EjSOC1

In the previous experiment, the expression level of *EjSPL*s in the apical buds showed the possibility that they may regulate the expression of the downstream flowering genes. It has been previously reported that some *SPLs* can directly bind to *LFY*, *AP1*, and *SOC1* promoters to regulate their transcription in *Arabidopsis*, and the specific binding site sequence is GTAC [35,51]. Therefore, in order to verify whether *EjLFY*, *EjAP1,* and *EjSOC1* can be regulated by *EjSPL*s, we cloned the promoters of *EjLFY*s, *EjAP1*s, and *EjSOC1*s from the genomic DNA of “JFZ” loquat, respectively. The lengths of these six promoter regions are all about 2000 bp (upstream of each *EjSPL* genes initiation codon ATG). We used the PlaceCARE website to perform cis-acting element prediction on the promoter regions of these genes; some binding site sequences (GTAC) were found in their promoter regions (Sequence S2).

**Figure 7:**
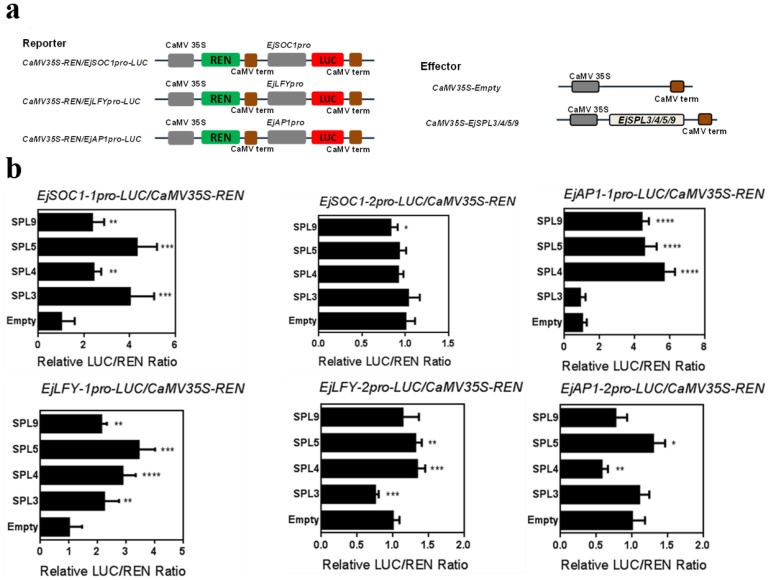
The activation of *EjSPL3*, *EjSPL4*, *EjSPL5* and *EjSPL9* on the expression of *EjLFY*, *EjAP1,* and *EjSOC1*. The activation effect was detected using dual luciferase system. (**a**) Vectors construction. (**b**) *EjSPL3*, *EjSPL4*, *EjSPL5,* and *EjSPL9* activated the *EjLFY*, *EjAP1,* and *EjSOC1* promoters. Error bars indicating SE from at least six biological replicates. The asterisk indicates a significant difference compared to the empty effector (Student’s *t*-test, * *p* < 0.01, ** *p* < 0.001, *** *p* < 0.0001, **** *p* < 0.00001).

We constructed the reporter vectors, in which, CAMV35S drives renilla luciferase (REN), and the *EjLFY-1pro*, *EjLFY-2pro*, *EjSOC1-1pro*, *EjSOC1-2pro*, *EjAP1-1pro*, and *EjAP1-2pro* drive firefly luciferase (LUC). *EjSPL3*, *EjSPL4*, *EjSPL5,* and *EjSPL9* were constructed into the effector vector driven by CAMV35S (Figure 7a). The two vectors were co-transformed into tobacco by transient expression by different combinations to analyze whether *EjSPL3*, *EjSPL4*, *EjSPL5,* and *EjSPL9* could transcriptionally activate the downstream genes promoters. From the luciferase signal, it was showed that *EjSPL3*, *EjSPL4*, *EjSPL5,* and *EjSPL9* obviously activated the expression of LUC, which was driven by the promoters of *EjLFY-1* and *EjSOC1-1*; and *EjSPL4*, *EjSPL5,* and *EjSPL9* remarkably activated the expression of LUC driven by the *EjAP1-1* promoter compared to the control (Figure 7b).

### 2.6. EjSPL3, EjSPL4, EjSPL5, and EjSPL9 Promote Flowering in Arabidopsis

**Figure 8:**
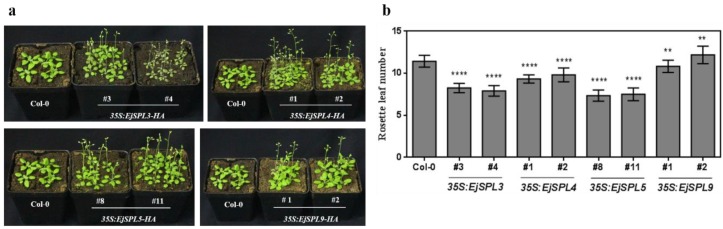
Overexpression of *EjSPL3*, *EjSPL4*, *EjSPL5,* and *EjSPL9* in *Arabidopsis* promotes early flowering. (**a**) Phenotype of overexpression transgenic lines and wild Col-0. (**b**) The rosette leaf numbers of overexpression transgenic lines and Col-0 in (**a**). Error bars indicating SE from three biological replicates. The asterisk indicates a significant difference compared to the Col-0 (*n* ≥ 15, Student’s *t*-test, ** *p* < 0.01, **** *p* < 0.00001).

To investigate the genetic functions of *EjSPL3*, *EjSPL4*, *EjSPL5,* and *EjSPL9*, overexpression vectors containing full length coding sequences of the four *EjSPL* genes were constructed and transformed into wild-type *Arabidopsis thaliana* (Col-0). The homozygous transgenic lines were screened out, and the flowering time and the expression of downstream flowering genes were analyzed in these transgenic lines. All transgenic lines with different genes showed early flowering phenotype compared to wild-type Col-0 (Figure 8a). Under the same growing condition, Col-0 had about 12 rosette leaves when flowering, while the transgenic lines with *35S: EjSPL3* and *35S: EjSPL5* had only seven to eight rosette leaves; *35S: EjSPL4* had nine to ten rosette leaves; *35S: EjSPL9* had 10 to 11 rosette leaves (Figure 8b). The expression levels of the related flowering genes including *AtAP1*, *AtLFY*, and *AtSOC1*, showed different degrees of improvement (Figure S1). There was no obvious different morphological characteristic of the above transgenic lines observed compared to that of Col-0, such as flower organs, leaf shapes, siliques, and cauline leaves. From the above results, *EjSPL3*, *EjSPL4*, *EjSPL5,* and *EjSPL9* all have the function of promoting flowering in *Arabidopsis*.

## 3. Discussion

To date, remarkable achievements have been made in the research of flowering regulation mechanism in plants, including photoperiod pathway, gibberellin pathway, autonomous pathway, aging pathway, vernalization pathway, ambient temperature pathway, and so on, which integrate each other to form a huge and complex regulatory network [52]. Among the above pathways, *SPL* transcription factors play a very important role in the control of flowering time. Our results show that all members of the *SPL* gene family contain the SBP-box domain, which was conserved in a certain degree in different species (Figure 1 and Figure 2). In this study, it was suggested that *EjSPL3*, *EjSPL4*, *EjSPL5,* and *EjSPL9* are involved in the regulation of flowering time in loquat and are highly conserved with their homologous genes. Differential expression levels and subcellular localization implied that they may have different patterns in regulating loquat growth and development.

Functional verification of *SPL3*, *SPL4*, *SPL5,* and *SPL9* homologous genes in other plants has also been reported. For example, overexpression of *SPL3* in *Arabidopsis* wild-type results in early flowering, and knocking out *SPL3*, *SPL4*, *SPL5*, and *SPL9* delays flowering time and produces more rosette leaves [13]. *MdSPL3* of apple encodes 189 amino acids and is localized in the nucleus; overexpressing it in *Arabidopsis* wild-type turns into fewer trichomes and promotes morphological differentiation, the *MdSPL3* to be highly expressed in the rosette leaves and stem of transgenic *Arabidopsis,* and finally significantly improves the expression of downstream genes (*AtFT*, *AtSOC1*, *AtLFY*, *AtAP1*, *AtFUL*) [53]. In citrus, overexpression of *CiSPL5* removed the binding site of miR156 in the 3′UTR, to *Arabidopsis* wild-type, leading to early flowering and fewer small rosette leaves [54]. *PtSPL9* is expressed in various organs, with the highest expression in stems, and showed a nuclear localization [55]. In grape, *VpSBP11* (a homolog of *AtSPL3/4/5*) encoded 170 amino acids, contained a highly conserved SBP domain, and its protein was localized in the nucleus with the transcriptional activity; overexpressing it (removed 3′ UTR, the binding site of miR156) into the *Arabidopsis* wild-type advanced flowering, and promoted the expression of *FUL*, *AP1*, *LFY,* and some other genes; and also, the phenotypic characteristics in transgenic plants were transformed [36]. Combined with our results, it is shown that the *SPL* genes are both functionally conserved and functionally differentiated during plant development.

The regulatory network of *SPL* is extremely complicated, and to date, the model plant *Arabidopsis* is the most thoroughly studied. Most of the *SPL* genes in *Arabidopsis* are targeted by miR156 and miR157 [14,56,57], and positively regulate downstream flowering genes directly, such as *SOC1*, *LFY*, *AP1*, *FUL*, and so on [35,51]. This regulation pattern has also been verified in other species [28,53,58,59]. In this study, we found some *SPL* binding sites (GTAC) in the promoter regions of *EjLFY*, *EjSOC1*, and *EjAP1* (Sequence S2); and the dual-luciferase reporter assays verified that EjSPLs activate their expression, in which, EjSPL3, EjSPL4, EjSPL5, and EjSPL9 effectively activated *EjLFY-1* and *EjSOC1-1* expression; and EjSPL4, EjSPL5, and EjSPL9 significantly improve the expression of *EjAP1-1* (Figure 7). In addition, we found that the expression levels of the floral meristem identity genes *AtAP1* and *AtLFY* were significantly different among different transgenic lines (Figure S1). These results suggested that the *SPL* genes in loquat may be coordinated with each other to act on different downstream genes to jointly regulate flowering.

There are also some novel regulatory models found in other plants. *SlSBP3* and *SlSBP15* of tomato are regulated by the activity of PROCERA/DELLA, which can activate the *SINGLE FLOWER TRUSS* (*SFT*) gene in the leaves and the *AP1/MC* in the shoot apex to promote flowering [60]. In the *Arabidopsis thaliana*, *SODIUM POTASSUIM ROOT DEFECTIVE1* (*NaKR1*) was found to respond to the changes in potassium, and it could regulate the expression of *FT* through the miR156-SPL3 module, which also affected the transport of FT protein in the phloem [61]. The nuclear factor *CmNF-YB8* is a direct upstream gene of cmo-miR156, which can directly bind to its promoter region. Silencing this gene led to down-regulation of cmo-miR156 and up-regulation of *CmSPL3*, *CmSPL5,* and *CmSPL9* [58]. Interestingly, *EjFT2* showed involvement in the process of flower bud differentiation in loquat [62]. In order to ascertain whether there is a similar pattern of flowering regulation in the *EjSPL* genes in loquat, we need more in-depth research.

## 4. Materials and Methods

### 4.1. Plant Materials and Growth Conditions

Cultivated loquat (*Eriobotrya japonica* Lindl.) was used in this study. Adult loquat trees were grown under natural conditions in the loquat germplasm resource preservation garden, South China Agricultural University, Guangzhou, China. The wild-type of *Arabidopsis*, *Arabidopsis thaliana* Columbia-0 (Col-0), was used for gene transformation. *Nicotiana benthamiana* was cultivated for the assays of transient expression. The seeds of the *Arabidopsis* Col-0 and tobacco used in this study were provided by Xingliang Hou. Both of *Arabidopsis* and tobacco were grown under long-day conditions (16 h light/8 h dark) at 22 °C in a controlled environment room. Samples (loquat tissues and *Arabidopsis* plants) for qRT-PCR were immediately frozen in liquid nitrogen and stored in an ultra-low temperature refrigerator at −80 °C until use.

### 4.2. Gene Cloning and Sequence Analysis

The coding region of *EjSPL3*, *EjSPL4*, *EjSPL5,* and *EjSPL9* were amplified through PCR using PrimeSTAR^®^ Max DNA Polymerase (TaKaRa, shiga, Japan) with specific primers (Table S1). The PCR products were connected with pGEM-T vector (Promega, Wisconsin, USA). Then the cloned products were sequenced and blasted with the other homologous sequences in the NCBI (https://www.ncbi.nlm.nih.gov/). DNAMAN 6.0, ClustalX 2.0.12, GeneDoc 2.7, and MEGA 6 software were employed for the amino acid sequences alignment and the construction of the phylogenetic tree of *SPL* proteins, respectively.

### 4.3. Gene Expression Analysis with qRT-PCR

The total RNA of loquat and *Arabidopsis* was extracted by EASYspin Plus plant RNA extraction kit (Aid lab, Beijing, China), and the cDNA was synthesized using PrimeScript^TM^ RT reagent Kit with gDNA Eraser (TaKaRa, shiga, Japan). Quantitative real-time PCR (qPCR) was performed using iTaq^TM^ universal SYBR Green Supermix (Bio-Rad, Hercules, CA, USA) in the LightCycler 480 (Roche, Basel Switzerland). *Ejβ-actin* [63] and *TUB2* (AT5G62690) were amplified as the internal control genes for normalization in loquat and *Arabidopsis thaliana*, respectively. Three technical and biological replicates were applied and data were analyzed with previous methods [64]. Semi-quantitative RT-PCR was employed for detecting the expression of exogenous gene in the overexpression lines of *Arabidopsis*.

### 4.4. Subcellular Localization Analysis

The full length coding sequences of *EjSPL3*, *EjSPL4*, *EjSPL5*, and *EjSPL9* without stop codon were separately cloned into the pGreen-35S-GFP vector [65]. These pGreen-35S-EjSPL-GFP or negative control vectors were transformed into the *Agrobacterium tumefaciens* strain *GV3101::psoup* by heat induction, and the transformed *Agrobacterium* cells were cultured and injected into epidermic cells of tobacco (*Nicotiana benthamiana*) leaves for transient expression following the previously described methods [66]. After about 48 h of injection, GFP signal was observed using the fluorescence microscope (Observer.D1, Zeiss, Jena, Germany).

### 4.5. Arabidopsis Transformation

The full length coding sequences of *EjSPL3*, *EjSPL4*, *EjSPL5,* and *EjSPL9* were alone cloned into pGreen-35S-6HA vector [67] to generate the overexpressed *EjSPLs*. Then, these recombined vectors were transformed into *Arabidopsis* Col-0 by the floral dip through *Agrobacterium*-mediated method [68]. Finally, T3 homozygous transgenic lines were screened using Basta.

### 4.6. Promoter Cloning and Analysis

The promoters of *EjLFY*, *EjAP1* and *EjSOC1* were cloned from the DNA of “JFZ” using PrimeSTAR^®^ Max DNA Polymerase (TaKaRa) with specific primers. Putative cis-acing elements in each promoter region were found in the PlantCARE database (http://bioinformatics.psb.ugent.be/webtools/plantcare/html/).

### 4.7. Dual-luciferase Reporter Assays

For the analysis of binding activity of EjSPL3, EjSPL4, EjSPL5, and EjSPL9 to the promoters of *EjLFY*, *EjAP1,* and *EjSOC1*, the above promoters were cloned into pGreenII 0800-LUC double-reporter vector, firefly luciferase (LUC), and Renilla luciferase (REN), and the full length coding sequences of *EjSPL3*, *EjSPL4*, *EjSPL5,* and *EjSPL9* without stop codon were cloned into pGreenII 62-SK effector vector [69].

The recombined effectors and reporters were co-transformed into tobacco leaves with different groups by *Agrobacterium*-mediated protocol, as described above. After about 48 h of transformation, we quantified the activity of LUC and REN using the dual luciferase assay kit (Promega) with appropriate modification [70] was quantified on the Luminoskan Ascent Microplate Luminometer (Thermo Fisher Scientific). The binding activity was calculated by the LUC to REN ratio. Each assay was made by at least six biological duplicates.

### 4.8. Data Analysis

All data analyses were performed by Excel and GraphPad Prism 6.01. Significance of differences between data was evaluated by Student’s *t* test.

## 5. Conclusions

In summary, the *Eriobotrya japonica* transcription factors EjSPL3, EjSPL4, EjSPL5, and EjSPL9 are speculated to participate in flower bud differentiation and flower organ development by activating the expression of *EjSOC1*, *EjLFY*, or *EjAP1*.

## Supplementary Materials

Supplementary materials can be found at https://www.mdpi.com/1422-0067/21/1/248/s1. Table S1; Sequence S1; Sequence S2; Figure S1.

## References

[1] Blazquez M.A., Weigel D. (2000). Integration of floral inductive signals in Arabidopsis. Nature.

[2] Amasino R.M., Michaels S.D. (2010). The Timing of Flowering. Plant Physiol..

[3] Fornara F., de Montaigu A., Coupland G. (2010). SnapShot: Control of flowering in Arabidopsis. Cell.

[4] Srikanth A., Schmid M. (2011). Regulation of flowering time: All roads lead to Rome. Cell. Mol. Life Sci..

[5] Yu S., Cao L., Zhou C.-M., Zhang T.-Q., Lian H., Sun Y., Wu J., Huang J., Wang G., Wang J.-W. (2013). Sugar is an endogenous cue for juvenile-to-adult phase transition in plants. Elife.

[6] Zuo Z., Liu H., Liu B., Liu X., Lin C. (2011). Blue Light-Dependent Interaction of CRY2 with SPA1 Regulates COP1 activity and Floral Initiation in Arabidopsis. Curr. Biol..

[7] Putterill J., Laurie R., Macknight R. (2004). It’s time to flower: The genetic control of flowering time. Bioessays.

[8] Wellmer F., Riechmann J.L. (2010). Gene networks controlling the initiation of flower development. Trends Genet..

[9] Wang J.-W. (2014). Regulation of flowering time by the miR156-mediated age pathway. J. Exp. Bot..

[10] Kardailsky I., Shukla V.K., Ahn J.H., Dagenais N., Christensen S.K., Nguyen J.T., Chory J., Harrison M.J., Weigel D. (1999). Activation tagging of the floral inducer FT. Science.

[11] Ferrandiz C., Gu Q., Martienssen R., Yanofsky M.F. (2000). Redundant regulation of meristem identity and plant architecture by FRUITFULL, APETALA1 and CAULIFLOWER. Development.

[12] Lee J.-H., Park C.-M. (2015). Integration of photoperiod and cold temperature signals into flowering genetic pathways in Arabidopsis. Plant Signal. Behav..

[13] Xu M., Hu T., Zhao J., Park M.-Y., Earley K.W., Wu G., Yang L., Poethig R.S. (2016). Developmental Functions of miR156-Regulated SQUAMOSA PROMOTER BINDING PROTEIN-LIKE (SPL) Genes in Arabidopsis thaliana. PLoS Genet..

[14] He J., Xu M., Willmann M.R., McCormick K., Hu T., Yang L., Starker C.G., Voytas D.F., Meyers B.C., Poethig R.S. (2018). Threshold-dependent repression of SPL gene expression by miR156/miR157 controls vegetative phase change in Arabidopsis thaliana. PLoS Genet..

[15] Jones-Rhoades M.W., Bartel D.P., Bartel B. (2006). MicroRNAs and their regulatory roles in plants. Annu. Rev. Plant Biol..

[16] Chuck G., O’Connor D. (2010). Small RNAs going the distance during plant development. Curr. Opin. Plant Biol..

[17] Klein J., Saedler H., Huijser P. (1996). A new family of DNA binding proteins includes putative transcriptional regulators of the Antirrhinum majus floral meristem identity gene SQUAMOSA. Mol. Gen. Genet..

[18] Cardon G.H., Hoehmann S., Nettesheim K., Saedler H., Huijser P. (1997). Functional analysis of the Arabidopsis thaliana SBP-box gene SPL3: A novel gene involved in the floral transition. Plant J..

[19] Yang Z., Wang X., Gu S., Hu Z., Xu H., Xu C. (2008). Comparative study of SBP-box gene family in Arabidopsis and rice. Gene.

[20] Tripathi R.K., Bregitzer P., Singh J. (2018). Genome-wide analysis of the SPL/miR156 module and its interaction with the AP2/miR172 unit in barley. Sci. Rep..

[21] Kropat J., Tottey S., Birkenbihl R.P., Depege N., Huijser P., Merchant S. (2005). A regulator of nutritional copper signaling in Chlamydomonas is an SBP domain protein that recognizes the GTAC core of copper response element. Proc. Natl. Acad. Sci. USA.

[22] Riese M., Hoehmann S., Saedler H., Muenster T., Huijser P. (2007). Comparative analysis of the SBP-box gene families in P-patens and seed plants. Gene.

[23] Salinas M., Xing S., Hoehmann S., Berndtgen R., Huijser P. (2012). Genomic organization, phylogenetic comparison and differential expression of the SBP-box family of transcription factors in tomato. Planta.

[24] Zhang L., Wu B., Zhao D., Li C., Shao F., Lu S. (2014). Genome-wide analysis and molecular dissection of the SPL gene family in Salvia miltiorrhiza. J. Integr. Plant Biol..

[25] Xie K., Wu C., Xiong L. (2006). Genomic organization, differential expression, and interaction of SQUAMOSA promoter-binding-like transcription factors and microRNA156 in rice. Plant Physiol..

[26] Lannenpaa M., Janonen I., Holtta-Vuori M., Gardemeister M., Porali I., Sopanen T. (2004). A new SBP-box gene BpSPL1 in silver birch (*Betula pendula*). Physiol. Plant.

[27] Li X.-Y., Lin E.-P., Huang H.-H., Niu M.-Y., Tong Z.-K., Zhang J.-H. (2018). Molecular Characterization of SQUAMOSA PROMOTER BINDING PROTEIN-LIKE (SPL) Gene Family in Betula luminifera. Front. Plant Sci..

[28] Cai C., Guo W., Zhang B. (2018). Genome-wide identification and characterization of SPL transcription factor family and their evolution and expression profiling analysis in cotton. Sci. Rep..

[29] Hou H., Li J., Gao M., Singer S.D., Wang H., Mao L., Fei Z., Wang X. (2013). Genomic Organization, Phylogenetic Comparison and Differential Expression of the SBP-Box Family Genes in Grape. PLoS ONE.

[30] Li J., Hou H., Li X., Xiang J., Yin X., Gao H., Zheng Y., Bassett C.L., Wang X. (2013). Genome-wide identification and analysis of the SBP-box family genes in apple (Malus x domestica Borkh.). Plant Physiol. Biochem..

[31] Zhang W., Li B., Yu B. (2016). Genome-wide identification, phylogeny and expression analysis of the SBP-box gene family in maize (*Zea mays*). J. Integr. Agric..

[32] Yamasaki K., Kigawa T., Inoue M., Tateno M., Yamasaki T., Yabuki T., Aoki M., Seki E., Matsuda T., Nunokawa E. (2004). A novel zinc-binding motif revealed by solution structures of DNA-binding domains of Arabidopsis SBP-family transcription factors. J. Mol. Biol..

[33] Wu G., Park M.Y., Conway S.R., Wang J.-W., Weigel D., Poethig R.S. (2009). The Sequential Action of miR156 and miR172 Regulates Developmental Timing in Arabidopsis. Cell.

[34] Schwarz S., Grande A.V., Bujdoso N., Saedler H., Huijser P. (2008). The microRNA regulated SBP-box genes SPL9 and SPL15 control shoot maturation in Arabidopsis. Plant Mol. Biol..

[35] Jung J.-H., Lee H.-J., Ryu J.Y., Park C.-M. (2016). SPL3/4/5 Integrate Developmental Aging and Photoperiodic Signals into the FT-FD Module in Arabidopsis Flowering. Mol. Plant.

[36] Hou H., Yan X., Sha T., Yan Q., Wang X. (2017). The SBP-Box Gene VpSBP11 from Chinese Wild Vitis Is Involved in Floral Transition and Affects Leaf Development. Int. J. Mol. Sci..

[37] Yu N., Niu Q.-W., Ng K.-H., Chua N.-H. (2015). The role of miR156/SPLs modules in Arabidopsis lateral root development. Plant J..

[38] Xing S., Salinas M., Hoehmann S., Berndtgen R., Huijser P. (2010). miR156-Targeted and Nontargeted SBP-Box Transcription Factors Act in Concert to Secure Male Fertility in Arabidopsis. Plant Cell.

[39] Ferreira e Silva G.F., Silva E.M., Azevedo M.d.S., Corazon Guivin M.A., Ramiro D.A., Figueiredo C.R., Carrer H., Pereira Peres L.E., Silveira Nogueira F.T. (2014). microRNA156-targeted SPL/ SBP box transcription factors regulate tomato ovary and fruit development. Plant J..

[40] Liu Y., Zhao Q., Meng N., Song H., Li C., Hu G., Wu J., Lin S., Zhang Z. (2017). Over-expression of EjLFY-1 Leads to an Early Flowering Habit in Strawberry (Fragaria x ananassa) and Its Asexual Progeny. Front. Plant Sci..

[41] Shikata M., Koyama T., Mitsuda N., Ohme-Takagi M. (2009). Arabidopsis SBP-Box Genes SPL10, SPL11 and SPL2 Control Morphological Change in Association with Shoot Maturation in the Reproductive Phase. Plant Cell Physiol..

[42] Yu S., Galvao V.C., Zhang Y.-C., Horrer D., Zhang T.-Q., Hao Y.-H., Feng Y.-Q., Wang S., Schmid M., Wang J.-W. (2012). Gibberellin Regulates the Arabidopsis Floral Transition through miR156-Targeted SQUAMOSA PROMOTER BINDING-LIKE Transcription Factors. Plant Cell.

[43] Chuck G., Whipple C., Jackson D., Hake S. (2010). The maize SBP-box transcription factor encoded by tasselsheath4 regulates bract development and the establishment of meristem boundaries. Development.

[44] Hou H., Jia H., Yan Q., Wang X. (2018). Overexpression of a SBP-Box Gene (VpSBP16) from Chinese Wild Vitis Species in Arabidopsis Improves Salinity and Drought Stress Tolerance. Int. J. Mol. Sci..

[45] Lin S., Sharpe R.H., Janick J., Janick J. (1999). Loquat: Botany and Horticulture. Horticultural Reviews.

[46] Esumi T., Tao R., Yonemori K. (2005). Isolation of LEAFY and TERMINAL FLOWER 1 homologues from six fruit tree species in the subfamily Maloideae of the Rosaceae. Sex. Plant Reprod..

[47] Liu Y., Song H., Liu Z., Hu G., Lin S. (2013). Molecular characterization of loquat EjAP1 gene in relation to flowering. Plant Growth Regul..

[48] Zhang L., Yu H., Lin S., Gao Y. (2016). Molecular Characterization of FT and FD Homologs from Eriobotrya deflexa Nakai forma koshunensis. Front. Plant Sci..

[49] Zhang L., Jiang Y., Zhu Y., Su W., Long T., Huang T., Peng J., Yu H., Lin S., Gao Y. (2019). Functional characterization of GI and CO homologs from Eriobotrya deflexa Nakai forma koshunensis. Plant Cell Rep..

[50] Jiang Y., Peng J., Zhu Y., Su W., Zhang L., Jing Y., Lin S., Gao Y. (2019). The Role of EjSOC1s in Flower Initiation in Eriobotrya japonica. Front. Plant Sci..

[51] Yamaguchi A., Wu M.-F., Yang L., Wu G., Poethig R.S., Wagner D. (2009). The MicroRNA-Regulated SBP-Box Transcription Factor SPL3 Is a Direct Upstream Activator of LEAFY, FRUITFULL, and APETALA1. Dev. Cell.

[52] Huijser P., Schmid M. (2011). The control of developmental phase transitions in plants. Development.

[53] Fan S., Zhang D., Gao C., Wan S., Lei C., Wang J., Zuo X., Dong F., Li Y., Shah K. (2018). Mediation of Flower Induction by Gibberellin and its Inhibitor Paclobutrazol: mRNA and miRNA Integration Comprises Complex Regulatory Cross-Talk in Apple. Plant Cell Physiol..

[54] Shalom L., Shlizerman L., Zur N., Doron-Faigenboim A., Blumwald E., Sadka A. (2015). Molecular characterization of SQUAMOSA PROMOTER BINDING PROTEIN-LIKE (SPL) gene family from Citrus and the effect of fruit load on their expression. Front. Plant Sci..

[55] Song C.N., Qian J.L., Fang J.G., Wang H.K., Qiu X.L., Zhang Z., Zhang X.Y. (2010). Cloning, Subcellular Localization and Expression Analysis of SPL9 and SPL13 Genes from Poncirus trifoliata. Sci. Agric. Sin..

[56] Gandikota M., Birkenbihl R.P., Hoehmann S., Cardon G.H., Saedler H., Huijser P. (2007). The miRNA156/157 recognition element in the 3′ UTR of the Arabidopsis SBP box gene SPL3 prevents early flowering by translational inhibition in seedlings. Plant J..

[57] Jung J.-H., Seo P.J., Kang S.K., Park C.-M. (2011). miR172 signals are incorporated into the miR156 signaling pathway at the SPL3/4/5 genes in Arabidopsis developmental transitions. Plant Mol. Biol..

[58] Wei Q., Ma C., Xu Y., Wang T., Chen Y., Lu J., Zhang L., Jiang C.-Z., Hong B., Gao J. (2017). Control of chrysanthemum flowering through integration with an aging pathway. Nat. Commun..

[59] Long J.-M., Liu C.-Y., Feng M.-Q., Liu Y., Wu X.-M., Guo W.-W. (2018). miR156-SPL modules regulate induction of somatic embryogenesis in citrus callus. J. Exp. Bot..

[60] Silva G.F.F., Silva E.M., Correa J.P.O., Vicente M.H., Jiang N., Notini M.M., Junior A.C., De Jesus F.A., Castilho P., Carrera E. (2019). Tomato floral induction and flower development are orchestrated by the interplay between gibberellin and two unrelated microRNA-controlled modules. New Phytol..

[61] Negishi K., Endo M., Abe M., Araki T. (2018). SODIUM POTASSIUM ROOT DEFECTIVE1 regulates FLOWERING LOCUS T expression via the microRNA156-SQUAMOSA PROMOTER BINDING PROTEIN-LIKE3 module in response to potassium conditions. Plant Cell Physiol..

[62] Reig C., Gil-Munoz F., Vera-Sirera F., Garcia-Lorca A., Martinez-Fuentes A., Mesejo C., Perez-Amador M.A., Agusti M. (2017). Bud sprouting and floral induction and expression of FT in loquat [*Eriobotrya japonica* (Thunb.) Lindl.]. Planta.

[63] Shan L.L., Li X., Wang P., Cai C., Zhang B., De Sun C., Zhang W.S., Xu C.J., Ferguson I., Chen K.S. (2008). Characterization of cDNAs associated with lignification and their expression profiles in loquat fruit with different lignin accumulation. Planta.

[64] Livak K.J., Schmittgen T.D. (2001). Analysis of relative gene expression data using real-time quantitative PCR and the 2-DELTADELTACT method. Methods.

[65] Lee L.Y.C., Hou X., Fang L., Fan S., Kumar P.P., Yu H. (2012). STUNTED mediates the control of cell proliferation by GA in Arabidopsis. Development.

[66] Sparkes I.A., Runions J., Kearns A., Hawes C. (2006). Rapid, transient expression of fluorescent fusion proteins in tobacco plants and generation of stably transformed plants. Nat. Protoc..

[67] Hou X., Zhou J., Liu C., Liu L., Shen L., Yu H. (2014). Nuclear factor Y-mediated H3K27me3 demethylation of the SOC1 locus orchestrates flowering responses of Arabidopsis. Nat. Commun..

[68] Zhang X., Henriques R., Lin S.-S., Niu Q.-W., Chua N.-H. (2006). Agrobacterium-mediated transformation of Arabidopsis thaliana using the floral dip method. Nat. Protoc..

[69] Hellens R.P., Allan A.C., Friel E.N., Bolitho K., Grafton K., Templeton M.D., Karunairetnam S., Gleave A.P., Laing W.A. (2005). Transient expression vectors for functional genomics, quantification of promoter activity and RNA silencing in plants. Plant Methods.

[70] Moyle R.L., Carvalhais L.C., Pretorius L.-S., Nowak E., Subramaniam G., Dalton-Morgan J., Schenk P.M. (2017). An Optimized Transient Dual Luciferase Assay for Quantifying MicroRNA Directed Repression of Targeted Sequences. Front. Plant Sci..

