# Supplementary material for: The Role of EjSPL3, EjSPL4, EjSPL5, and EjSPL9 in Regulating Flowering in Loquat (Eriobotrya japonica Lindl.)
Source: Int J Mol Sci. 2019 Dec 30;21(1):248. doi: 10.3390/ijms21010248 (PMC6981807; doi:10.3390/ijms21010248)
Supplement: Supplementary file 1 [file ijms-21-00248-s001.zip › Supplementary Data.docx]

## Supplementary Data

**Table S1** Primers for all experiments.

| Usage | Name | Sequences | |
| --- | --- | --- | --- |
|  |  | Forward primer (5'-3') | Reverse primer (5'-3') |
| Full length | *EjSPL3* | ATGATGTGTTGTTGGAGTGGGTCTACG | ATTAGACGTGCCTCCATGTCCATGGTA |
|  | *EjSPL4* | ATGGAGGGCAAGAACTTTGAA | TTATCTGATCTGGCAATGCTT |
|  | *EjSPL5* | ATGGAGGGCAAAAACTTTGA | TTATCTGATCTGGCAATGCT |
|  | *EjSPL9* | ATGGAAATGGGCTCGAGCT | TTAAAGTGACCAGTGCATCT |
| qRT-PCR | *Ejβ-actin* | GGATTTGCTGGTGATGATGC | CCGTGCTCAATGGGATACTT |
|  | *EjSPL3* | CAGCAATGTAGCAGGTTTC | CATGGTATTCCGATGACG |
|  | *EjSPL4* | GGATGACTACTCGGATGAAG | CAACCAAATCAGCCTCAC |
|  | *EjSPL5* | TTGTGATTGTTTCCGGGACT | GTTCGCCTGTGCTCTTGC |
|  | *EjSPL9* | TCCAATCACTTTCCGACCAC | GTTGCTGCCTCCTTCCCT |
|  | *EjAP1* | CTCGGATGCTTTGCTGCTTG | AGTACGTTCAGGGTTGGTCC |
|  | *AtTUB2* | ATCCGTGAAGAGTACCCAGAT | AAGAACCATGCACTCATCAGC |
|  | *AtAP1* | CATGGGTGGTCTGTATCAAGAAGAT | CATGCGGCGAAGCAGCCAAGGTT |
|  | *AtLFY* | ACGCCGTCATTTGCTACTCT | CTTTCTCCGTCTCTGCTGCT |
|  | *AtSOC1* | AGCTGCAGAAAACGAGAAGCTCTCTG | GGGCTACTCTCTTCATCACCTCTTCC |
|  | *AtFUL* | ATGATGGAACTCCGTTGTCG | TTCATGAGAAATCATTACCAAGATATG |
|  | *AtmiR172b* | TTTCTCAAGCTTTAGGTATTTGTAG | TCGGCGGATCCATGGAAGAAAGCTC |
|  | *AtFT* | CTTGGCAGGCAAACAGTGTATGCAC | GCCACTCTCCCTCTGACAATTGTAGA |
|  | *AtFD* | GCTCACTTGCAGGCAGAAAA | CCTTTTCTCTTTCCGGGTCT |
| Subcellular localization and overexpression | *pGreen-35S/GFP-EjSPL3-F* | GTCGACGGTATCGATAAGCTTATGATGTGTTGTTGGAGTGG | |
|  | *pGreen-35S/GFP-EjSPL3-R* | TCCCCCGGGCTGCAGGAATTCATTAGACGTGCCTCCATGTCC | |
|  | *pGreen-35S/GFP-EjSPL4-F* | GTCGACGGTATCGATAAGCTTATGGAGGGCAAGAACTTTG | |
|  | *pGreen-35S/GFP-EjSPL4-R* | TCCCCCGGGCTGCAGGAATTCTCTGATCTGGCAATGCTTG | |
|  | *pGreen-35S/GFP-EjSPL5-F* | CCCAAGCTTATGGAGGGCAAAAACTTT | |
|  | *pGreen-35S/GFP-EjSPL5-R* | CGCGGATCCTCTGATCTGGCAAT | |
|  | *pGreen-35S/GFP-EjSPL9-F* | GTCGACGGTATCGATAAGCTTATGGAAATGGGCTCGAGCT | |
|  | *pGreen-35S/GFP-EjSPL9-R* | TCCCCCGGGCTGCAGGAATTCAAGTGACCAGTGCATCT | |
| Dual-lucliferase reporter assay | *pGreenⅡ0800-EjLFY-1PRO- F* | GTCGACGGTATCGATAAGCTTggaagggagtatgtaggcg | |
|  | *pGreenⅡ0800-EjLFY-1PRO- R* | CGCTCTAGAACTAGTGGATCCtattttccacaactccgca | |
|  | *pGreenⅡ0800-EjLFY-2PRO- F* | GTCGACGGTATCGATAAGCTTcagcattacacgcctcttaag | |
|  | *pGreenⅡ0800-EjLFY-2PRO- R* | CGCTCTAGAACTAGTGGATCCactttccacaactccacacag | |
|  | *pGreenⅡ0800-EjAP1-1PRO- F* | GTCGACGGTATCGATAAGCTTggacggcgaagatgtaaagc | |
|  | *pGreenⅡ0800-EjAP1-1PRO- R* | CGCTCTAGAACTAGTGGATCCagagcttgcctttgttggaga | |
|  | *pGreenⅡ0800-EjAP1-2PRO- F* | GTCGACGGTATCGATAAGCTTggatataaatcttacccctcagag | |
|  | *pGreenⅡ0800-EjAP1-2PRO- R* | CGCTCTAGAACTAGTGGATCCtgattaaatattaatggactctcagtta | |
|  | *pGreenⅡ0800-EjSOC1-1PRO- F* | GTCGACGGTATCGATAAGCTTtcttaggtccgattctcgc | |
|  | *pGreenⅡ0800-EjSOC1-1PRO- R* | CGCTCTAGAACTAGTGGATCCtttttatgatttaattatattaatttcctt | |
|  | *pGreenⅡ0800-EjSOC1-2PRO- F* | GTCGACGGTATCGATAAGCTTaaaatcatcaactttcatgtcgt | |
|  | *pGreenⅡ0800-EjSOC1-2PRO- R* | CGCTCTAGAACTAGTGGATCCtttttgatgatttaatttttaattaattt | |
|  | *pGreenⅡ62SK-EjSPL3- F* | CGCTCTAGAACTAGTGGATCCATGATGTGTTGTTGGAGTGG | |
|  | *pGreenⅡ62SK-EjSPL3- R* | GTCGACGGTATCGATAAGCTTTTAATTAGACGTGCCTCCAT | |
|  | *pGreenⅡ62SK-EjSPL4- F* | CGCTCTAGAACTAGTGGATCCATGGAGGGCAAGAACTTTG | |
|  | *pGreenⅡ62SK-EjSPL4- R* | GTCGACGGTATCGATAAGCTTTTATCTGATCTGGCAATGCTT | |
|  | *pGreenⅡ62SK-EjSPL5- F* | CGCTCTAGAACTAGTGGATCCATGGAGGGCAAAAACTTT | |
|  | *pGreenⅡ62SK-EjSPL5- R* | GTCGACGGTATCGATAAGCTTTTATCTGATCTGGCAATGCT | |
|  | *pGreenⅡ62SK-EjSPL9- F* | CGCTCTAGAACTAGTGGATCCATGGAAATGGGCTCGAGCT | |
|  | *pGreenⅡ62SK-EjSPL9- R* | GTCGACGGTATCGATAAGCTTTTAAAGTGACCAGTGCATCT | |

**Sequence S1** Cloned *EjSPL* genes sequence and deduced amino acid sequence

***EjSPL3***

**1 ATGATGTGTTGTTGGAGTGGGTCTACGGCAGGGCCATGTTGTCAGGTAGATAATTGCACTGCTGACTTGAGTGATTTAAAGCAATACTACCGCCGC**

**1 M M C C W S G S T A G P C C Q V D N C T A D L S D L K Q Y Y R R**

**97 CACAAGGTCTGTGATGTTCATGCCAAGGCTCCGTCGATAGTCGTCGGTGGATCCCGGCAGCGTTTTTGCCAGCAATGTAGCAGGTTTCATAGCCTA**

**33 H K V C D V H A K A P S I V V G G S R Q R F C Q Q C S R F H S L**

**193 TCAGAGTTTGATGACAGTAAAAGGAGTTGTCGCAGGCGATTAGCTGGACACAATGAGCGGCGTCGGAAAACGTCATCGGAATACCATGGACATGGA**

**65 S E F D D S K R S C R R R L A G H N E R R R K T S S E Y H G H G**

**289 GGCACGTCTAATTAA**

**97 G T S N ***

***EjSPL4***

**1 ATGGAGGGCAAGAACTTTGAAGGAAGGCAAACATGGAAAGAGAAGGCGAAGAAGGATGTCGAGGAAGTAGAGGATGACTACTCGGATGAAGAAGAG**

**1 M E G K N F E G R Q T W K E K A K K D V E E V E D D Y S D E E E**

**97 ACCAGTGGTGGTGGCGGAGGGTTGATGCTGAGATTTGAAGAAAATGAGAGGCAGAAGAAAGCTGCTGCAGCTGGAAGAAGAGGATCTGGTGGCGGA**

**33 T S G G G G G L M L R F E E N E R Q K K A A A A G R R G S G G G**

**193 GGTGGAGGTGGGATAGCGCAACCGTTTTGTCAAGCGGAGAGGTGTGAGGCTGATTTGGTTGATGCAAAGAGGTACCACCGCCGGCATAAGGTTTGT**

**65 G G G G I A Q P F C Q A E R C E A D L V D A K R Y H R R H K V C**

**289 GAGCTTCATTCTAAGGCAGCTGTTGTGATCGTTTCCGGGATTCAACAGCGGTTTTGCCAGCAATGCAGCAGGTTCCATGAGCTAATTGAATTTGAC**

**97 E L H S K A A V V I V S G I Q Q R F C Q Q C S R F H E L I E F D**

**385 GAAGCAAAGAGAAGCTGTCGCCGGCGTTTGGCAGGACACAATGAACGACGCCGCAAGAGCTCAGGCGAACCTTATGGAGAAGGATCGAGCCGGAGA**

**129 E A K R S C R R R L A G H N E R R R K S S G E P Y G E G S S R R**

**481 GGGGTTGGTCACCAGTACAAAGAAAGTCAGACTGGATATCAGATCACACCCCCAGGAAACTCTTCTTCCAAGCATTGCCAGATCAGATAA**

**161 G V G H Q Y K E S Q T G Y Q I T P P G N S S S K H C Q I R ***

***EjSPL5***

**1 ATGGAGGGCAAAAACTTTGAAGGAAGGCAAACATGGAAAGAGAAATCGAAGAAAGATGTTGAGGAAGTAGAGGATGACTACTCGGACGAAGAAGAG**

**1 M E G K N F E G R Q T W K E K S K K D V E E V E D D Y S D E E E**

**97 ACCAGTGGCGGTGGTGGAGGGTTGATGCTGAGATTTGAAGAAAATGAGAGGCAGAAGAAAGCTGCTGCAGCTGGAAGAAGAGGATCTGGTGGTGGA**

**33 T S G G G G G L M L R F E E N E R Q K K A A A A G R R G S G G G**

**193 GGTGGAGGTGGAGATGGGATTTCACAGCCACCTTGTCAAGCGGAAAGGTGCGGGGCTGATTTGGTTGATGCAAAGAGGTACCACCGCCGGCATAAG**

**65 G G G G D G I S Q P P C Q A E R C G A D L V D A K R Y H R R H K**

**289 GTTTGCGAGTTTCATTCGAAGGCAGCTGTTGTGATTGTTTCCGGGACTCGGCAGCGGTTTTGTCAGCAATGCAGCAGGTTCCATGAGCTAATTGAA**

**97 V C E F H S K A A V V I V S G T R Q R F C Q Q C S R F H E L I E**

**385 TTTGATGAAGCAAAGAGAAGCTGTCGCCGGCGTCTGGCAGGACACAATGAGCGACGCCGCAAGAGCACAGGCGAACCTTATGGAGAAAGCTCAAGC**

**129 F D E A K R S C R R R L A G H N E R R R K S T G E P Y G E S S S**

**481 CGGAGAGGGGTTGGTCACCAATACAAAGAAAGTCAGACTAGATATCAGATCACGCCCCCAGGAAACTCTTCCTCCAAGCATTGCCAGATCAGATAA**

**161 R R G V G H Q Y K E S Q T R Y Q I T P P G N S S S K H C Q I R ***

***EjSPL9***

**1 ATGGAAATGGGCTCGAGCTCTATGACCGAGTCAGGGAGCTCTTCCTCCTCTTCGCCGCCCAACTCCTCCGCTGAGTCACTCAACGGCTTAAAATTC**

**1 M E M G S S S M T E S G S S S S S S P P N S S A E S L N G L K F**

**97 GGCCGGAAAATCTACTTTGAGGATGGGGGTTTTGGAGCTCTGCACAAATCATCATCCGGGTCTGCTGCTGGGTCTTCCTCCGCCGGGGCTACGCCG**

**33 G R K I Y F E D G G F G A L H K S S S G S A A G S S S A G A T P**

**193 CCCAAGAAGCAAAGGGGCGGCGGAAATTTGGGTCAGCCGCCGCGGTGTCAGGTGGAGGGCTGCGAGGTAGATCTGAGTGGTGCCAAAGCTTACTAT**

**65 P K K Q R G G G N L G Q P P R C Q V E G C E V D L S G A K A Y Y**

**289 TCCAGGCACAAAGTCTGTGGCTTGCACTCTAAAACCCCCACTGTCATTGTTGCTGGTCTTGAACAGAGGTTTTGCCAACAGTGTAGCAGGTTTCAT**

**97 S R H K V C G L H S K T P T V I V A G L E Q R F C Q Q C S R F H**

**385 TTACTTCCTGAATTTGACCAAGGAAAACGTAGTTGTCGTAGACGCTTGGCTGGGCATAATGAGCGTCGTAGAAAGCCACAACCAGGATCCATACTG**

**129 L L P E F D Q G K R S C R R R L A G H N E R R R K P Q P G S I L**

**481 TCTACGCGTGGCAGACTTTCTTCGTCTCTCTACGAAAACAGCAACAGAATTGGAAGCTTTCTGATGGACTTCACTGCATACCCAAGGCCTTCTGGG**

**161 S T R G R L S S S L Y E N S N R I G S F L M D F T A Y P R P S G**

**577 AGGGATGCATGGACAACAACGAGAACGTCTGAGCGAGCACCTGCTAATCAAAATGCCAATGATGCAGGGAAGTTTCTTCAACAGCCGTGGCAGAGC**

**193 R D A W T T T R T S E R A P A N Q N A N D A G K F L Q Q P W Q S**

**673 AACTCTGAGATTTCTACATCCGGCTTTTACCTACAAGGTTCAGCAGGCGGGACTAGTTATCCTGGTCCTGGAATTCCTCCAGGAGAATGCGTCACA**

**225 N S E I S T S G F Y L Q G S A G G T S Y P G P G I P P G E C V T**

**769 GTAGTCACAGACTCAAGCTGTGCTCTCTCTCTTCTGTCAAATCAGCCATGGGGCTCTCGAAACCGAGTATCAGGTGCTGGGATGAATTCCTTGATG**

**257 V V T D S S C A L S L L S N Q P W G S R N R V S G A G M N S L M**

**865 AACACTCAAGGGGTACCTGTGGCTCAACCAGTCCCTCATTCTGCGACCTCCAATCACTTTCCGACCACTACGTGGGGTTTCAAAGGAAATGAAAAT**

**289 N T Q G V P V A Q P V P H S A T S N H F P T T T W G F K G N E N**

**961 GGTAGCAGCTCACAAGAGATGCTTCCAGATCTGGGTCTCGGTCAAATCTCGCAGCCGCTTAGCAGTCAGTACTCTGGTGTGCTGGAGCTGTCTCAA**

**321 G S S S Q E M L P D L G L G Q I S Q P L S S Q Y S G V L E L S Q**

**1057 CAGGGAAGGAGGCAGCAACACATGGAACTCGGACACACCAGGGGCTATGACTCCACCAGTCAGCAGATGCACTGGTCACTTTAA**

**353 Q G R R Q Q H M E L G H T R G Y D S T S Q Q M H W S L ***

**Sequence S2** Promoter nucleotide sequences of *EjLFY*, *EjSOC1* and *EjAP1*. The specific binding site sequence, GTAC, is marked by red and boxed. Translation start site (ATG) was shown in yellow box.

***EjAP1-1PRO*(Eri000407.1)**

GACGGCGAAGATGTAAAGCCATACTTATAGTTGGTGGGTATGACATGGCCATAAACGCTCTTTTCAATTTGTAGGGTGGTTGGGGTCCAATTGAATCATTCAATTACAACTCTATTACTACTAATGGATGCCTGGTTGGTGCGTCCTCCCTATTATGCTTTGGTTAGGGAAACAATTTTGAACCCCATATAATTATTCTAGATTTTTTTTTTTTTTTTTTTTTTTTTTTTTTTTTTTTTTTTTTTTTGTGGGTCGAGATAGTCATGCTAGATGATCTACGTAAGAAAATAAGATAAATGTTAAAAGCAAAGTGATGATCAGTTAGAAGAAAGAGACAGCACATCTTAAAATCTGTTTGAGATTACTAAACAAATAATTAACTAAAACTTAATTGAGAATCTAAAATTGCTTCTGACATGCCACTCAGTACTACGGTCTGGTGGTATTCCTCTTCACTTGAAAGTGAGAGGTCTTAGGTTCGAATCTCGTGGATGATACGTTGCCCATTGTGTGGTTTAGCCGAACTCCCCCTCCACTTAGTGTAAAAATATCGATGTATTAAAAAAAATGCTTCTAAAATAGCTAAAAGTCTTTTCTAACAATAAAAAATGTTTTTTTAACTACATTTGAAAGAAAACGTTAATATTTGTAGGTTATAAAAGCATTTTCTAGAGAAAGACATATCATTCTTCCCTGTAGCACTAGGATTTTTTTTTTATAATAATATTATACATGTTTTTAATAAAAACACTTCTAACAAAAATAGTCTCAACTTCCTTAAACTCTTTATTATTCATATATATATATATATATAACACGATGTTAACATGACATGCATACATATATTTCACTAACAATATGGTAACCAAATGATCCCCTCCTCATTTTATGTAGGAAAAATGAGTTAGCTTAATCATTTTCAGTTCATAAGAGACTAACAGTGTATATCATCAATATATTGAATAATATAAACCGTAAAGGAGATAGAGATTAACAATTAAATATTGTTTTCGTGTCAAACTGATCATGAACACATTGTTTTCTTTTTTTTTGGACAAAGATCATGAACACATTGTTGAAATGTGGGACACCAACGAGCAGTATAATTCCTCTGGCCAAAAACAAACAAATTTTCAACCTACCAATACTAATGGGGTCAAATCACAGTAATTCTCCCTGTATAAAATGTGGCACTTTGCGTACGTACGTTCGCCGGTTACGAGTCAAGCAATAAGGCAATGGTAACGCTGAAGGCCGTCCGAACAATGCATGCCTGAGACTCTATGAGTCCGAATAGAAGCCAGTAAGTAGTGATCTACACCAGTGTGAAAGAAAAATAAAATTGTGGTTCCAATTACTAGCCCTGTTGGAAAGAGAGAAAACGACTACATAGTGTACCTCAACCAATCAGAAATCGACAACACTTTCTTAGAAATCCCACCGGTTTTCCCAAAACCACTTGTATTCACAAAACCGCTTTATCTGTCATCAAATCAAATGCATGTATAGATAGCTACTAGCACGTACACTTTCTCAGACACCTTCAGATATTTGGCTAGAAGAAGAAATTTTACATAAATTAAAATTCCCATTAATTTATAACAGTGTTTCTTTCTTTTTGCATATAAATAGCACAGCTTAGTATTTCAGTTTCTGGGTTGTCTTTCTTTTTTTGGTTGTTTGGGTTTTTTGAGAAAGAAAATCAGAGAACAAGCAGTTGAAAGAGTTGAGTGGCAACTGAGAGTCCATAAGTATTTAATCATGGGGAGAGGTAGAGTTCAGCTTAAGCGCATAGAGAACAAGATCAACAGGCAGGTGACTTTTTCCAAAAGAAGAACTGGGCTGCTGAAGAAGGCTCATGAGATCTCTGTCTTGTGTGATGCTCAAGTTGCTGTGGTTGTCTTCTCCAACAAAGGCAAGCTCTATG

***EjAP1-2PRO*(Eri030184.1)**

GGATATAAATCTTACCCCTCATAGAGCAAGAGAAACAAAATACAAGAAAAAAAGGGAGGAACAAACACCTTACCTCTATTGAATAAGAAAATACAAGAAAAAAAAGGGGGGATATAGACCTTACCTCTCTTGAAAAAGAAAATACAAGAAAAATAATGGGTGGACATAGGCCCAACCCCTCTATAAACCTAAAAGGTAGGAATTTGCAAGACAAGCTGCAAAACGAAAAGGAAAATCCAAAAAGATTAGGTAAAAAAGAAAATCACACATCAAGAAGGGGAGACAAACTTGTAGGCTAGCATACGCAAGAAATCTTAGTGCAGAAGAGAAAAAATCTCCATAGGAGGAGCGGCATGTCAAACAAGTGAAGACGAGATAAGCTATAAATTGGCTAATTTGTCAGCAACTGCATTCCCTTCTCGAAATGTATAAGAGCAACAAAATACCATGTTCTGCAATCAGAAAATACAATTTTTCCAACGTGTTTGGAGCGACCAAGGAGGAGAGAAAGATCTATAAGCAAAACAAGATATCATACTAGAAGAGTCACTTTCAAGCCATAAATTTTGCCAACCCCGTGCGTGGGCTAACTCCACAATAAGGATGATAGCATGAAGCTCCGCATAAAAAGAAGTACGATGCCCCAAACTTAGGGAAAACTACCAAGAAAATAACCCGCAGAATCTCGGAAAACCCCAGTAAAAGCCACATAACCCAGGTTACCTTTAGCAAGGTCATCAGTACCAAGAAAATGGTGAAGGATGTCAAAGAACATGGATTATAGATAGAGTTTTACAAGACTCAACCGAGATTACAAGAGAGACTAAAAGTTGCTTACCCAAAATACCCCGTCCGTTACCAGGGTGAAAACTCCAACTTGCCTAATCCATACCGAAATGAATGGCAAAGGGGTGAGAAGGAATAAGGCTTACTTTCAAACTTCACTTTATTACGCATCTTCCAAATAGCCATTAGCAAGAAAAACCATGAAGCTAGCCAAACATTACAAAGTTGTGGAGAAAACCACTTTGACACAAAAGCAAGCGACAAATATGAGAGAGAACCTGTAAGCAGAAAAATAGTTCCAAATTAGGTAGCTAGCCAACACCAAGCCCATTGTGCAAATTCACAACTAAAAAATAAGTGCTCAATAGATTTCGAATTTTTGTAACATAGTTGGCATATTAGAACCAACGGAATGCCCTGTCGCTAAAGTTCATCCTCCGTTGGAAGCTTTTTGAAAGAATCTTTCAAACTAAAATAGAGTAGCGAGGTGGAATGAAAGGACGCCAAATAATGGAGGCGCAACTCTTAACAGAAAATCAATGACGAACAATTTCATAGCCATCAGATAACAAAATGACGAACAATTTTATTTTTTAAGTTATTAACTTTTTAGCACATATCTCACTATTTGTATAATAGCACGTGATGTACTATTTTGTGTGCTAATCACACTGAAAAATCTCTCATGCCAGAGACTAATGGGGGTCAAATCACGGTAATTTTCCCTAAACAAAAAGTGGCACTGTGCATACGCACGTCAGCTGGTTACGCGTCGAGAAAAAAAAGTAACGGTAAAGCTGAAGGTCCGTACAATGCATACCTGAGGTTCTTGAGTCCGGCAGGAAGCCGGTAATAGTGACCTACACCGGTAGGTAAGAAAGATAAAATCGAGGTTTCTTTTTCCTGGCCCCGTTGGAAAGAGAGAAAACGACTACATAGTACACCTCAGCTAATCAGAAGTCGACAACACATCCTTTAAAACCTCACCGGTTTTCCCAAAACCACTTTGATTCACAAAAACCGCTTTATCTATCATCAAATCAAAAGTCTGTAGAGCTAGCTACTAGCACATATACTTTCCCAGGCACCTTCACATATTTGGCTATAAGAAGAAACTTTGCATAAATTAATATTCCCATTAAATTGTAACTATATTTCTTTCTTTTTTGTATATAAATCGACAGCCTACCAGTTCATTTTCTGGGTTGTCTTTCTTTTTCTGGGGGTTTGGGTTGTTTGGTTTTTTTGAGAAAGAAAATCAAAGAACAAGCAGTTGAAAGAGTGAGTGGTAACTGAGAGTCCATTAATATTTAATCAATG

***EjLFY-1PRO*(Eri022269.1)**

GGAAGGGAGTATGTAGGCGGAGATGCAGCTGATCGTCACCCCCTTTGATGTGGGCATCGAAAGTACAAAAGCAGCCTTTTCAGTTCTGAGACACCTGCCCAACTCTTGTACTAGCCCAGTTGTCCTGACAGTTTTTCAATTTTATTTTTGTACAGGGAACAGTAAACACAGTGATGACATTGTGTACGTGTTCGTGAAAGTACATTTATGCCCTCGCATGCTTGACCTGTTGGGGCAATGAAATATCCAAGAATCTCTACAGAGATAGAGACATGGGGTTGTGTCTGTCACGTTGCCCTAGACCGACTGCTGGAGCGTGATCGATCATGATGAGTCTGATGATGATATGATGGTCGAAATCAAATTACCATGAATTACACAAACACCAATCTTAATCTTAAACATTCCATTATACCAAAATTAGGTTTTTTTTTTCTTTCTTATGAATAATTTCTCACCGTCAATTTTAATCTGAACTGTGAAGGTTTTTTATTACCAAGTTTTGCAAAAAAGGAAAGAAAAAAAAACTGTTTGTGTGTTTGAATCATGTCACATATTTTGGACATTTAGATGTTGGTTTTGTCATGAGTAGGAGACCAAAGGATACAAGTAGCTAGACAGCCAAGAAAACAAGAAGCAAACAAGAAAGGAAGTTGGGACAAGTTACTTCTATTTAGCACTTTGAGGAGATTTGTCTTTCCTTCATAAAAGGGTTGATTCAGGGCATGTCAAACCTTGGTAAGGTTCTTCGCTTTGCATCGAATTAAACCACATGCTCCATCCTTTTTGTGGGCTTCCGTCAATTCATTATTGAATGGGTAGTAGGTACAATTTTGAATGTTTTGCTTATGTGCAAAGTAACAAATATTATAATTGGATCGTTTGATCACTTTTTAGTCATTCATTGAATGATAAATCACTACAAGTGAAGGAGATACACGACTTAAATAACAAAAAGGAAATAGGATTTCGATATGAATGTCGTCTATTAACCAGTTTTTTGGAAATTCTTTTTATGATAATTGGTAGGAATTTTATATCTTTATTTCCTTCTTAGCAAATAATTTTTGTTTCCACAAGGTATATATCGTAATGTCTTTCTATAGGATCACAGGTCTTTTTATTAGCTTGTGTTTATGGTGCACAATTTCATGGAATGTAGGTAGCTGTTATGATTAATTCAATATCAATTCATCCTTTTTTTTTTCTTTCAGGAGTTTCATAATTGGACATTGAACCCATATCATGGGTAAATTATTATTATTTTTTTAAATCATATTATTTTCGGTGTATAGGATATATCGATATTTTATTATCTAGACCTTGGTCGTTCGTTTCTTAACCAAGGATCTAATTATAAAAAACAGTGGTTATAGACTATCCGGAAGTATATAAAAAAAGTCCCTTAACTTTTATGCTCAGCCATACTAACACGGGAAATCTATCTTAGCAAAATTGAGGTTACGTTAATGTCAACGAAGTTCTATGGTGTTCTGCCCTTCGTGTAGAAGAGGGCTTGATATGTCTGCAAATAAAAGTTACTCTTGTTTTTTGTAAGTAATTTATGCGTATGCATCAGACTGTTATTTGTAAAAATAAAAAGAAACTTCAACAAGAAAAGTCGGACTCTTTTTTGGCTCATTTTCTCTTACAGACTCCCCTACATTCGATGGAGTTTATATTTGAATTTAATGATGACATCATAGAAATTAGATACTATATGAACCAAATCCAACCTTAAGGTCAACTCAAATCTATCAGAGGCATGCATAGATCTTCATTGGCTAGACCTAACAAACATAACAAGTTAAAGAAGTTCCATGGGTAGTGGGACCAATTGGATGGACCCTAACAAGTTAAACGACACCTTCGTTTTGGTTGTTACAATTTGGTTGGGTTTGGAAGTGTCAAGTTTGAATACAAGTATACGAACTCCAGTATGTCAGTTCCATAGGAAACACTCGTACAAAACAAAAGGGTAGTTTCGGAAGTATAAGAAAAATAGTCTTGCTTTTAGTTTTATAGGAACAGAACACTACAAGCTCCCACAGAAAATAGTCTCTGTTCACACTGTGCTGTGCGGAGTTGTGGAAAATAATG

***EjLFY-2PRO*(Eri007397.1)**

ACTGATTGAATCGCATTATTGCTAGCTCATTGTGAGGCTAACCCCATCCCCTCCCTCTTAGTGTAGATAATATCGTTTATTAAAAAAAAATCATTTGATAACTTATTTCATCACATTGTTATCCACGTGCGAAAAACCTTTTTTATAACTAACATTACATGCCTCTTAAGATGTCTTCAACATGTTTAAAAATAAAGAAATTGAATCACATTATTGCTAGCTGATTGTGAAGTACTAATTAAAAAAAAAAGCACCAAAAAGTAAATTATTAAAGAAATATTATTAAAATGACAAAAATGTCTCTACCTTATTTGATGTATTATTTTGCGCCTTTGAAGTTTTTTGTTTTGGGGATATTTTTGTCCACATATTTTTGTTGAATCATGGTGACACCAAATCACTATTCACATTTTCCATTCTCTTTATATATATACTAGCCTCCATGCACGCCCTGACGCGCGTGCGGAAGACCCTTTTATAATGACAGCATTCTACGCATGACTTATATAATTTAAGTGTATTTATTTACGTGGATTTGAAAAATGAATTAAACATTAAAAAAATGAATGTAACTTTGTCATATATTTCATCGAAGCAAAATATCTTTGTACACAACATTTCTTGTGAAGATTCCCATAAATTAACAAACATACAAAACACATAGGAAAACAACAGCTTCAACAAAATAAAAGGGCCAAAATACCCCTCTGCCAAAAAATAGTCCAAAACTGGCCCAAATCATATTTCAAAATTTGCAAGCGTAAACTAGTCCAAAAAAAAAAGTCCACTGTGCAGCACTGTGCACCTTTGCCTCTAGCTTTTGTGATGACAAAAATACCCTTGGATAATCTTAAACATTATGTTTTGCTGGTTGTAAATTTTTTTTTGGCGAGGGGTATTATCATCCAATTATTTTTGTCGAAGCTGGTGACACCAAAGTTTTGTCTGCATGCATAGACTCAAAACCACTGTTTTGCACTGTTTATCACTGTTTTGCACCGTTGACCTTTGCTCCGGCTTTCTATATAAAAGATATATATATATTTTTTTTTCATTCCTCTTTATAGGTGAATATATATATTTTCCAAAAGTTACTGTAAGATATATCATAATTTTTATCCTCTAAATCTCGCTGGTGGTTTCTTGACCAAAGATTTAATTACAAAAAACACTGGCGTATAGGCTACCTCGGAATATATTAAAAAATAAACTTTTACGCTCGAGTCTCGACCATACTAACACATTCTAGCTTAGCGAAATCGAGGTTATGTTAATGTCAACGAAGTTCTATGGTATTCGTGCCTTCGTATGGAAAAGGGTTTGATGTGTTTGCAAATAAAAACTACTCATGTTTTTTTTGTAAGTAATTTATGCATATGAATGGATCAGACTATTATTTGTATAAATGGAGAAAATTTTCAATAAGAAACTCAAAACCCTTTCTTGGTCCATTCTTTCTCACAAACTCCACTTCTTTCAGCTTTACTCATTGGGATTTAGGGTAGAATAATGACTCTATAAAAATTGTACGAACTTTTGAATAACTTGTTTTACATGTCTAATCACGTAATACCGCTACTAAAATTTCTCTAGTAAGAATGACCATGTAGAATCCAACCTTAAGTTCGACCAAAACAAAAATCCAACCTTAAATTTCAACTCAAATCTATGACAGCCATGCATAGACCTTCATAGGCTAGAGCCAACAGACATAACAAGTTAAAAGTTCATTGGGTAGTGGGACCAACTGGATGGACCCTAAAAAGTTAAACGACACCTTCAGTATGATTGTTATTATTTGGTTGGGTTTGGAAGTGTCAAGTTTGAATACAAGTATACGAACCCCGGTTTGTTAGTACCTCGGGAAACACTCGTACAAAACACAAGGGTAGTTTCGGAAGTATAAACAAAAATCGTCTTGCTTTTATAGGAACAGAACACTACAAGCCCCCCCAAGAAAATAGTCTGTTCACATTGTGCTGTGTGGAGTTGTGGAAAGTATG

***EjSOC1-1PRO*(Eri012338.1)**

AAAATCATCAACTTTCATGTCGTTTAATTTATAAAATTTTATCTACATATTTAGTTATTTAGCAATATCCCTTCTATAATTTAATCACAAACTTTTAATTATTTAATTAAATTTTTTTGGCTTTAAAAAAGTAAAGTAAACAAAACTGGGGAAAAGGGCAAAATGTGTTGTGCCAGACAAGGTGGGGGCCCGTGGGGACTACACCCCTAAGTCTGACAGGCAAATTAATGCTATAGGCTTGTTGCGTTTTCGACTTCTGATTGGCTGGAGGCCTACGTAGTCGTACGGACGATGTTGATGTATTGCTAACATTGTACTGTCTTTGCCGGTTTTAACTCTGCCCCTCCCATATTTTATGCTTTTTTTTTCTTTTGTTTTTTTTTGCCCTTTTTCTTTGCTTTCTGTTATGGGTGCCATTTTGTAGTTTATTGTCCATCACAAAAAACAAAATAAAAATCAGTGCATTAATAACAAACAATGATAAAATTAATATAAAAATATAATGTGCAGGGGGTGTATAATGTTCAGAATAAACCTACGTTGACAAGAAGAAAGACTTTACATATGTTTATAAATAATTGAGTTACTTTTTTTATTACTAATTAATTTTACGACGACATTTTAATATTTTTCGGTGGTTAGTATCTTCTTTAATCCACTTTGTCTTAAGGTCAAATTATTCCTCTCCTCCCACTGCAGGAACAATACATAATAAACAATGATAACATTAAATATAAAAAGCGCACCAACGCAGTACCTCAGTGCACTCTCATATTTGTACATCTCTCTCTTCGGTTTTTCTTTAGAGAACTTTCATCAAACAGGTAGTTTTAAGTTCCTTGCTTTCTCATAGTACTTGCCTTTTGTGTCCAGAATTTCTTTTTCTTGTGTGTTTTGTGGGTTTACCAAAACAAGCAAAGGATGAGGCATTTTTGCACACAACCTTTGATTTTTATATTATTATAGTATATTTTACGTTTCTTTTTCAAGGTGGGGTTTTAAGGAGAGAAGGATAGAAAAAACAAGAGAAAAAAGGGTAGGTGTGTTGGTTGCCTAAGGTCAATCATCAATCTCCACCCTCCATGTTTCTCTATAATTTCCTTCCTTTCTGGGTTTTTTAGTCTCCCCCCATCACTCTCTCTCTCTCTCTCTCTCTCTCTCTCTCTCTCTCTCTCTCCCTCCTCTTTTTTCTTTCTTTCTTTGGCATCTTTTTCTTCTCATATTTGTCCATAGCTTTTTCTGGGTTAGTTTGCATATTTGGTAAATTCGCTCAGTACCCTCTTCCACTACTCTGAAGAAGATCTTCTCTTTTTCTTTGAATCAGTCATTCACGAAACACCCTCCTCCTCGACCTTCTCCTCCTCCTCCTCGACCTCTCCCTCCCTCCCTCCCTCCCTCCCTCTCTCTCTCTCTCTCTCTCTCTATATATATATATATGCGCACATATATGTGCTTTTTTGGGGTGTAGTCCAAGTCCTTGCTCAAAAACTTGTGAGAAAAGAGCTCTCGCTTTCCACTTTTGGCTATTTGGTGATAATTTCGTAAGACTTACTTATTCCCTTAAGACTCTCTCTCTCTCTCTCTCTCATCCACTTTTTCTTTGTCTGTTTTTGTTTACTCTTAATTTTAATTTATGCACTCCTCGTTTGCTTACACAAATTATATCTATATGTGTATATATTGATTGCTTGCCAACCGTTTTGAACTGAACAGGATCTATTGCCTTCCACCTGATCTATACCATCATTCCAACTCACACAAAGAGCTTTTTGGGTTAATTTTGCTTATTTGGTAAGATTTAATTATCTTCTTCTTCTTTTCGTTATTATCATATACGTATATATCTCTTTTGTACACATTGGGATTTTTTTCAAGTTTTTGTCTGATGATTTCTGCAGAGACCAAAAAAAAAAAAAAAAAAAAAGAACTGATCTCAGAGCTTTCATTTTCTTGTGTCAAAAGGGTGGAAGGAAATTAATTAAAAATTAAATCATCAAAAAATG

***EjSOC1-2PRO*(Eri023104.1)**

TCTTAGGTCCGATTCTCGCCAAAGACAAATTTGAATCACATTATTGCTAACTTATTGTGTGGCTAAGAGTAAATAATATCGATTGTTAAAGAAAAAAAATTCAGTAGATACTAAACAATAATAATAGGGGTAATGCTAGAGAGATAAAAATTTTAAACTAAACTTGCAAACCAAATAATATGTTATTAATAAAAAATAAGCACGTTAATCAATGGTTAAGTAATAATCTAATAATCAACAATCACATTATTTAGTTTGTAAATTTAATTTAAAAAATTTATTTTCCTTAAAACATCATCTAAAAAATATTAATGGAATAACGTACCAACCCAGAAACTCAGTAGCTTTCTTTAGTTTTTCTTCTGAGAACTTTCATCAAACAAGCACTTTTATGTTCCTTGCTTTCTCCATTTCACAAAGAAAGTTCCTCGCTTTCTCGTAGTATTTGCCTTTTGTGTCCACAATTTCTTTTTCTGGTGTGTTTTGTGGGGTTACCAAAGCAAGCAAAGGATGAGACATTCAACCTTTGATTTTTATATTGTTAAAGTGTATTTTACTTGATAGGCTTCGAGTGTCCCCCTTCCTAACGGACTACTAGAGTACAACGGATTAGTGATACTATTTTTTTTATACAATTTTTCACATGTTTTGTAGGGTATATGTTTAATATATTTTAACGATCTAAATTGTTTATATTTTAGAAATTGTTCATAAATTATTTTTATAAAAAATAAGATAAATTCAAAACCATTAAGACATTCATTTATAATGAAGGAAATGGACGAATACTGTTTTATAAAGAAACCTAAATTGAATCTAATGGTCATATAGTTTCAGATTTCAATGATTTTTGGTACATATGATATTTTTTAGAAGACCTAAAAGATAAACATTTCGAATTATTAAAATATGTTAGTAAACCTACAAAAATGTGTGTAAAAATAATGGTGTCACTATTTAATGTTTCCTTTTCAAGGTGGGGTTTGAAGAGAGGGGATTTGGATCCTCTCCTAAGCTAATAGAGAGGATCCAGATCCGAAGATAGGATGGATGGAAGAAGAAGAAAAATAAGGCAAGAAAAAAATTAAAGAAAAAGGAAAGGTAGGTCTCTTGTTTGCCTCTGCTTACCCCAAGTAAAAGGTCAATCAACTCCACCCTCCCTCCTTATAATTTCCTTCCTTTCTTGGTTTTTTTGTATTTTCCCTCTCCCCTCTCGTTTTTCTTTCTTTCTTTGGCATCTTCTTCCTCTCATATTTGTCCATTACTTTTTTCTGGGTCTGTTTGCATATTTGGTAAATTTGCTCATTAAACTTTTCGACTACTCTGAAGAAGATTTTCCTTTTCTTTGAAATCAATCAATCAATCACAAAACACCCACCTCCCTCCTCTCTCTCTCTCTCTCTCTCTCTCTCTCTCTCTCTCTCTATATATATATATATATATATATAATATGCAAATATATGTGTGTTTTCAGGGTGGAATCATAGTCTTTGCTAAAAAAACTTAGAGAAGAGAGCTCTCTCTTTCCACTTTTAGTATTCCTTTGCCCCTCTCTCTGTCGCATCCACTTTCTTTGGCTGTTTTTGTTTTATTCTTCATATATATCTTCATTTTAATTTATGCACTCCTCCTTTGTTTACACAAATTATATCTATCTATATGTGTGTGTATATATATATATATATTGATTGCTTGCAACCCCTTTGAATTGAAGAGGATCATAGCCTTCCACCTCATCTATACCATCATTCCAACTCACAAAACCTAAAAAAAATAAAAAAGCTTTCTGGGTTAATTTTGCTTATTTGGTAAGATTTAATCATCTTCTCTTCTTCTTTTAGTTATTTTCATATATCTCTCTCTTTTGTACACATTGGTTTTTTTTTTTTAATCAAGTTTTTGTTTTAATGATTTCTGCAGAGACAAAAAAAAAAAAAAAAAACTGATCCCAGAGTTTAATTTTCTTGTGTCAAAAGGGTAGAAGGAAATTAATATAATTAAATCCTAAAAAATG


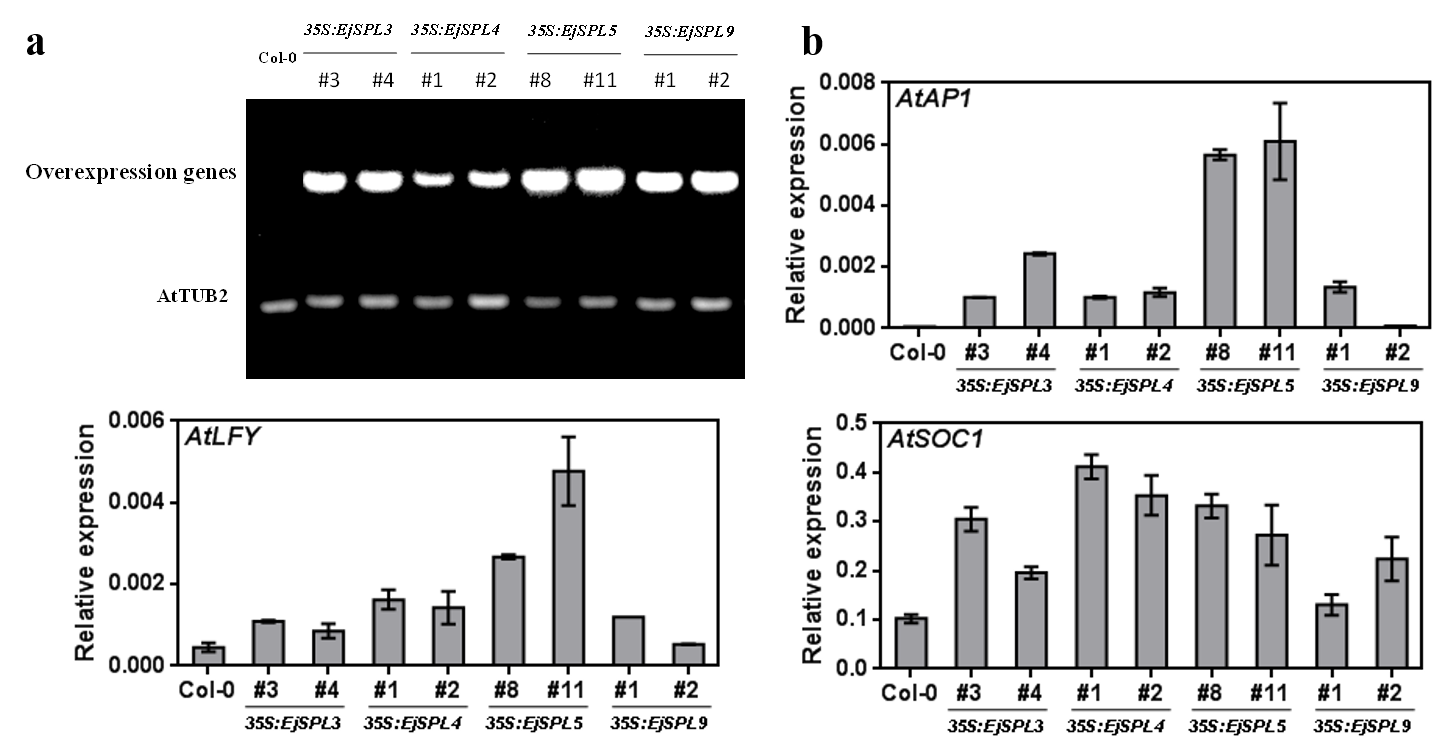


**Figure S1** Overexpression of *EjSPL3*, *EjSPL4*, *EjSPL5* and *EjSPL9* in *Arabidopsis* promotes early flowering. (a)Semi-quantitative of *EjSPL3*, *EjSPL4*, *EjSPL5* and *EjSPL9* in 14-day-old transgenic *Arabidopsis* seedlings. *Arabidopsis* *TUB2* (*AtTUB2*) severed as an internal control. (b) Relative expression of some downstream related genes of *Arabidopsis thaliana* in 14-day-old overexpression transgenic lines. Error bars indicating SE from three biological replicates.
